# Application of smog chambers in atmospheric process studies

**DOI:** 10.1093/nsr/nwab103

**Published:** 2021-06-15

**Authors:** Biwu Chu, Tianzeng Chen, Yongchun Liu, Qingxin Ma, Yujing Mu, Yonghong Wang, Jinzhu Ma, Peng Zhang, Jun Liu, Chunshan Liu, Huaqiao Gui, Renzhi Hu, Bo Hu, Xinming Wang, Yuesi Wang, Jianguo Liu, Pinhua Xie, Jianmin Chen, Qian Liu, Jingkun Jiang, Junhua Li, Kebin He, Wenqing Liu, Guibin Jiang, Jiming Hao, Hong He

**Affiliations:** State Key Joint Laboratory of Environment Simulation and Pollution Control, Research Center for Eco-Environmental Sciences, Chinese Academy of Sciences, Beijing 100085, China; Center for Excellence in Regional Atmospheric Environment, Institute of Urban Environment, Chinese Academy of Sciences, Xiamen 361021, China; College of Resources and Environment, University of Chinese Academy of Sciences, Beijing 100049, China; State Key Joint Laboratory of Environment Simulation and Pollution Control, Research Center for Eco-Environmental Sciences, Chinese Academy of Sciences, Beijing 100085, China; Aerosol and Haze Laboratory, Beijing Advanced Innovation Center for Soft Matter Science and Engineering, Beijing University of Chemical Technology, Beijing 100029, China; State Key Joint Laboratory of Environment Simulation and Pollution Control, Research Center for Eco-Environmental Sciences, Chinese Academy of Sciences, Beijing 100085, China; Center for Excellence in Regional Atmospheric Environment, Institute of Urban Environment, Chinese Academy of Sciences, Xiamen 361021, China; College of Resources and Environment, University of Chinese Academy of Sciences, Beijing 100049, China; State Key Joint Laboratory of Environment Simulation and Pollution Control, Research Center for Eco-Environmental Sciences, Chinese Academy of Sciences, Beijing 100085, China; Center for Excellence in Regional Atmospheric Environment, Institute of Urban Environment, Chinese Academy of Sciences, Xiamen 361021, China; College of Resources and Environment, University of Chinese Academy of Sciences, Beijing 100049, China; State Key Joint Laboratory of Environment Simulation and Pollution Control, Research Center for Eco-Environmental Sciences, Chinese Academy of Sciences, Beijing 100085, China; State Key Joint Laboratory of Environment Simulation and Pollution Control, Research Center for Eco-Environmental Sciences, Chinese Academy of Sciences, Beijing 100085, China; Center for Excellence in Regional Atmospheric Environment, Institute of Urban Environment, Chinese Academy of Sciences, Xiamen 361021, China; College of Resources and Environment, University of Chinese Academy of Sciences, Beijing 100049, China; State Key Joint Laboratory of Environment Simulation and Pollution Control, Research Center for Eco-Environmental Sciences, Chinese Academy of Sciences, Beijing 100085, China; State Key Joint Laboratory of Environment Simulation and Pollution Control, Research Center for Eco-Environmental Sciences, Chinese Academy of Sciences, Beijing 100085, China; College of Resources and Environment, University of Chinese Academy of Sciences, Beijing 100049, China; Beijing Convenient Environmental Tech Co. Ltd, Beijing 101115, China; Key Laboratory of Environmental Optics and Technology, Anhui Institutes of Optics and Fine Mechanics, Chinese Academy of Sciences, Hefei 230031, China; Key Laboratory of Environmental Optics and Technology, Anhui Institutes of Optics and Fine Mechanics, Chinese Academy of Sciences, Hefei 230031, China; State Key Laboratory of Atmospheric Boundary Layer Physics and Atmospheric Chemistry, Institute of Atmospheric Physics, Chinese Academy of Sciences, Beijing 100029, China; Center for Excellence in Regional Atmospheric Environment, Institute of Urban Environment, Chinese Academy of Sciences, Xiamen 361021, China; College of Resources and Environment, University of Chinese Academy of Sciences, Beijing 100049, China; State Key Laboratory of Organic Geochemistry and Guangdong Provincial Key Laboratory of Environmental Protection and Resources Utilization, Guangzhou Institute of Geochemistry, Chinese Academy of Sciences, Guangzhou 510640, China; Center for Excellence in Regional Atmospheric Environment, Institute of Urban Environment, Chinese Academy of Sciences, Xiamen 361021, China; State Key Laboratory of Atmospheric Boundary Layer Physics and Atmospheric Chemistry, Institute of Atmospheric Physics, Chinese Academy of Sciences, Beijing 100029, China; Center for Excellence in Regional Atmospheric Environment, Institute of Urban Environment, Chinese Academy of Sciences, Xiamen 361021, China; College of Resources and Environment, University of Chinese Academy of Sciences, Beijing 100049, China; Key Laboratory of Environmental Optics and Technology, Anhui Institutes of Optics and Fine Mechanics, Chinese Academy of Sciences, Hefei 230031, China; Center for Excellence in Regional Atmospheric Environment, Institute of Urban Environment, Chinese Academy of Sciences, Xiamen 361021, China; College of Resources and Environment, University of Chinese Academy of Sciences, Beijing 100049, China; Key Laboratory of Environmental Optics and Technology, Anhui Institutes of Optics and Fine Mechanics, Chinese Academy of Sciences, Hefei 230031, China; Center for Excellence in Regional Atmospheric Environment, Institute of Urban Environment, Chinese Academy of Sciences, Xiamen 361021, China; Shanghai Key Laboratory of Atmospheric Particle Pollution and Prevention, Department of Environmental Science and Engineering, Fudan University, Shanghai 200438, China; College of Resources and Environment, University of Chinese Academy of Sciences, Beijing 100049, China; State Key Laboratory of Environmental Chemistry and Ecotoxicology, Research Center for Eco-Environmental Sciences, Chinese Academy of Sciences, Beijing 100085, China; State Key Joint Laboratory of Environment Simulation and Pollution Control, School of Environment, Tsinghua University, Beijing 100084, China; State Key Joint Laboratory of Environment Simulation and Pollution Control, School of Environment, Tsinghua University, Beijing 100084, China; Center for Excellence in Regional Atmospheric Environment, Institute of Urban Environment, Chinese Academy of Sciences, Xiamen 361021, China; State Key Joint Laboratory of Environment Simulation and Pollution Control, School of Environment, Tsinghua University, Beijing 100084, China; Center for Excellence in Regional Atmospheric Environment, Institute of Urban Environment, Chinese Academy of Sciences, Xiamen 361021, China; College of Resources and Environment, University of Chinese Academy of Sciences, Beijing 100049, China; Key Laboratory of Environmental Optics and Technology, Anhui Institutes of Optics and Fine Mechanics, Chinese Academy of Sciences, Hefei 230031, China; College of Resources and Environment, University of Chinese Academy of Sciences, Beijing 100049, China; State Key Laboratory of Environmental Chemistry and Ecotoxicology, Research Center for Eco-Environmental Sciences, Chinese Academy of Sciences, Beijing 100085, China; State Key Joint Laboratory of Environment Simulation and Pollution Control, School of Environment, Tsinghua University, Beijing 100084, China; State Key Joint Laboratory of Environment Simulation and Pollution Control, Research Center for Eco-Environmental Sciences, Chinese Academy of Sciences, Beijing 100085, China; Center for Excellence in Regional Atmospheric Environment, Institute of Urban Environment, Chinese Academy of Sciences, Xiamen 361021, China; College of Resources and Environment, University of Chinese Academy of Sciences, Beijing 100049, China

**Keywords:** smog chamber, simulation, wall effect, secondary aerosol, ozone

## Abstract

Smog chamber experimental systems, which have been widely used in laboratory simulation for studying atmospheric processes, are comprehensively reviewed in this paper. The components, development history, main research topics and main achievements of smog chambers are introduced. Typical smog chambers in the world, including their volumes, wall materials, light sources and features, are summarized and compared. Key factors of smog chambers and their influences on the simulation of the atmospheric environment are discussed, including wall loss, wall emission and background pollutants. The features of next-generation smog chambers and their application prospect in future studies of the atmospheric environment are also outlined in this paper.

## Teaser

This review summarizes the key features of smog chambers and their application in atmospheric process studies, bringing new insights into development directions for next-generation smog chambers.

## INTRODUCTION

Air pollution and climate change are two of the most important ecological environmental problems that humanity must confront. Serious air pollution events have taken place in developed countries, such as the ‘London smog’ and ‘Los Angeles smog’, and are taking place in many developing countries, for example the ‘haze’ in China and India. Polluted air poses a major threat to health and ecosystems. Understanding the mechanism and evaluating the environmental impact of air pollution are crucial for policy making with regard to air pollution control and related scientific research.

In recent years, the rapid urbanization and economic growth in developing countries, such as China and India, have led to high emissions of various pollutants from coal combustion, motor vehicle exhaust and various industrial emissions, and resulted in high concentrations of fine particles (PM_2.5_, particulate matter with aerodynamic diameter less than 2.5 μm), SO_2_, NO_x_, NH_3_ and volatile organic compounds (VOCs). The cocktail of high concentrations of these pollutants, or so-called highly complex air pollution, has given rise to frequent haze pollution episodes. Despite the high intensity of primary emissions, secondary inorganic and organic species dominated PM_2.5_ during haze formation in terms of both mass and number concentrations [1–4]. Under these highly complex air pollution conditions, synergistic effects between pollutants may cause the ‘explosive growth’ of secondary aerosol and complex nonlinear relationships between secondary aerosol and its precursors [5–7]. Meanwhile, new particle formation (NPF) has been frequently observed under high pollution conditions [8–10]. How highly complex air pollution influences NPF, especially the growth of newly formed particles [11], remains highly uncertain. Simulating the chemical process under these highly complex air pollution conditions is critical for understanding haze chemistry and for further decision making.

This serious air pollution prompted strict air pollution controls, and remarkable achievements have been made in some developing countries. In China, a new air quality standard was set in 2012, in which the PM_2.5_ concentration was included for the first time. In September 2013, the state council released a clean air action plan and began to take practical actions to reduce the primary emissions of both gas pollutants and particle matters from thermal power plants, industry, on-road vehicles, etc. [12]. In the past five years, the air quality in eastern China has improved [13–17], with contributions from both meteorological condition changes [13,15] and emission controls [13,14]. Despite the 30%–50% decrease in annual mean PM_2.5_ across China over the 2013–2018 period [15,18,19], some gas pollutants showed decreasing trends, such as SO_2_ [13], while others showed increasing trends, such as NH_3_ [20] and O_3_ [18,19,21]. Emission inventory studies reported consistent changes in the corresponding pollutants. Zheng *et al.* [12] estimated the relative change rates of China's anthropogenic emissions between 2010 and 2017 to be −62% for SO_2_, −17% for NO_x_, +11% for non-methane volatile organic compounds (NMVOCs), +1% for NH_3_ and −27% for CO. The different change rates of pollutants also resulted in changes in atmospheric processes and particle compositions. Taking Beijing as an example, a much larger decline in sulfate than nitrate and ammonium led to a rapid transition from sulfate-driven to nitrate-driven aerosol pollution during the wintertime [22]. These phenomena highlight the urgency of understanding aerosol and ozone pollution in a changing atmospheric environment.

Field observation, laboratory study and numerical simulation are the three main approaches to investigating the physical and chemical processes in the atmosphere and their impact on our environment. Each of them has both advantages and disadvantages in studying the atmospheric environment. For example, field observation studies the real atmosphere directly but without reproducibility, and studies the contribution of different factors together but cannot isolate one factor and study it independently; laboratory study usually investigates atmospheric processes under well-controlled conditions but there is always the problem of whether it represents the real atmosphere; numerical simulation is helpful for integrating complex processes together, but its input and verification rely on laboratory studies and field observations. No single method can be omitted in the study of the atmospheric environment. For laboratory study, the smog chamber is well known for its ability to set experimental conditions close to those in the real environment and can also be carefully controlled. In this review, we summarize the application of smog chambers in studying the atmospheric environment in China and all over the world, discuss the factors influencing chamber simulations, and propose perspectives on future development of smog chambers.

## SMOG CHAMBER: ATMOSPHERIC PROCESS SIMULATOR

**Figure 1.**
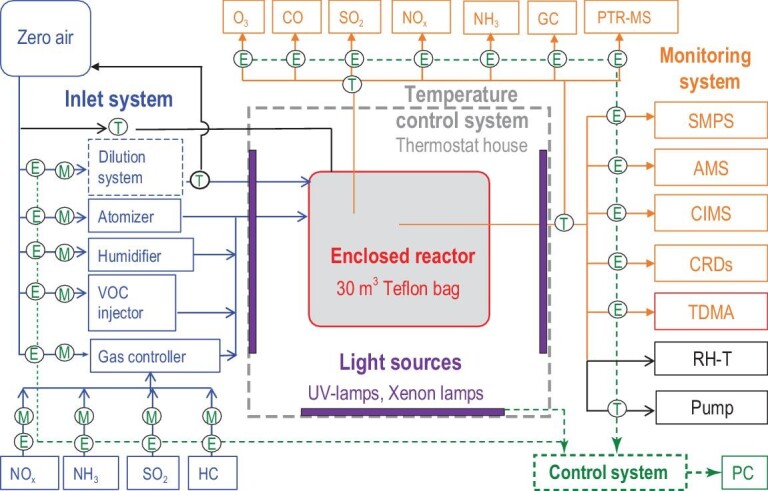
Schematic of the indoor chamber (taking the RCEES chamber as an example).

In general, a smog chamber is a reactor that simulates atmospheric processes under well-controlled conditions. A smog chamber can either be an outdoor chamber located on top of a building or a bracing structure, or an indoor chamber located in a building. The sizes of outdoor smog chambers are usually larger than indoor ones due to there being fewer space limitations. However, controlling the temperature and repeating an experiment are more difficult in outdoor smog chambers due to the significant greenhouse effects and the changing weather conditions. Typical indoor and outdoor smog chambers around the world are summarized in Tables S1 and S2 in the online Supplementary Data, respectively. They usually contain an enclosed space where the concentrations of pollutants and reaction conditions such as the temperature, relative humidity (RH) and irradiation can be controlled. Taking the indoor chamber in the Research Center for Eco-Environmental Sciences, Chinese Academy of Sciences (RCEES-CAS) as an example [23], a schematic of the indoor chamber is displayed in Fig. 1. Atmospheric processes are simulated in this enclosed space and monitored with a series of instruments. Chambers have been utilized to investigate atmospheric chemical mechanisms governing secondary aerosol formation.

A smog chamber experimental system usually contains the following parts: an enclosed reactor, inlet system, light sources, temperature control system, monitoring system and auxiliary system.

### The enclosed reactor

The reactor of a smog chamber can be made of Teflon film, stainless steel, Pyrex (borosilicate glass), quartz and so on. Teflon is the most widely used material for constructing the reactor of smog chambers, due to its good chemical stability and transmittance. A Teflon reactor also allows the operation of the smog chamber in static-state mode, i.e. simulating the reaction without adding continuous flow into the reactor, due to the fact that a Teflon reactor can collapse, to some extent, to adjust its volume with continuous sampling. Stainless steel has the advantage of reducing particle loss due to its good conductivity. The pressure in a reactor made of stainless steel can be adjusted to simulate atmospheric processes at different altitudes. Quartz could be a good material for constructing a reactor, but experience in building large reactors with quartz is still lacking. The pros and cons of different materials can be found in Finlayson-Pitts and Pitts [24].

The reactor volume of smog chambers varies from <1 m^3^ to hundreds of m^3^. To better simulate the real atmosphere, overly strong wall effects should be avoided. To minimize the wall effect, one can enlarge the volume of the reactor and choose inert wall materials. A large reactor volume can reduce the surface to volume ratio (S/V) and can also allow a bigger sampling volume for the monitoring instruments. However, the reactor volume is usually limited by many factors, such as the laboratory space, budget and so on. Besides, a larger volume will also increase the difficulty of maintaining homogeneous conditions in the reactor, which is usually crucial for accurately evaluating chemical mechanisms. To overcome this shortcoming in achieving homogeneous conditions, a mixing fan or mixing fans are usually placed in reactors with large volume. These fans will also help to increase the cleaning efficiency when flushing the reactor, and they can be either on or off during the reaction. In addition, mixing the reactor with fans will also increase wall deposition. In some smog chambers, the fans can mix the reactor at different speeds, aiming to strike a balance between homogeneity and wall deposition.

### Inlet system

To simulate the real atmosphere, different gases and aerosols need to be introduced into the reactor. The background clean air is usually the main component in the reactor. It can either be generated from purified air, which is usually called zero air, with concentrations of aerosol and gas pollutants as low as possible, or be made up of a mixture of nitrogen and oxygen. The air cleanliness is critical in studying chemical mechanisms, especially for experiments carried out with atmospherically relevant concentrations of pollutants. After cleaning and filling the reactor with background clean air, gas pollutants are usually introduced into the chamber from standard gas bottles at known concentrations or generated from liquid samples with a known volume through temperature-controlled tubes. Aerosols can be introduced into the smog chamber by different methods according to their physicochemical properties. The atomizer is widely used to generate aerosols, with a diffusion drier to remove water or organic solvent [25,26]. Besides atomizers, powder diffusers can also be used to generate aerosols from powder samples [27]. Some smog chambers also introduce pollutants from emission sources directly, such as vehicles, biomass burning and plants [28–30]. Special designs for the inlet, such as the provision of temperature control and dilution, are usually needed.

### Light sources

To simulate photochemical processes, light sources are needed in the smog chamber. For an outdoor chamber, direct solar irradiation has always been utilized, which has the advantage of consistency with real atmospheric conditions. Indoor chambers must use artificial irradiation to simulate photochemical processes. As summarized in previous publications [24,31], different types of lights, including mercury lamps (black lights), sun lamps, and xenon or argon arc lamps, have been used in different chambers. These light sources have different intensities and spectra. The most widely used light sources are UV lights with a peak wavelength of ∼350 nm (efficient for the photolysis of NO_2_) and a good UV cutoff at ∼300 nm. UV lights cannot represent the visible light in solar irradiation, however, and these visible wavelengths may be important for photolysis [32] and some aging processes [33]. To better simulate solar irradiation, arc lights are also used in some smog chambers. The spectrum of arc lights is closer to solar irradiation than UV lights, and was found to generate more secondary organic aerosol (SOA) compared to UV lights with similar amounts of VOCs consumed [34]. The irradiation intensity varies in different chambers, and can be adjusted in both indoor chambers (by turning on different numbers of lights) and outdoor chambers (by adjusting the irradiation area). The irradiation intensity is usually characterized by the NO_2_ photolysis rate (*J*_NO2_), which has been reported to be from 0.1 min^–1^ to higher than 1 min^–1^ in different smog chambers (Table S1). Constant and diurnally varying light conditions also have an impact on O_3_ formation [35]. Therefore, light conditions should be carefully characterized and taken into account to constrain the photoreactions.

### Temperature control system

Temperature is one crucial parameter that influences most reaction processes. Due to the introduction of irradiation, the temperature in the reactor will increase to unrealistic values without a cooling system. Besides, reactions occurring at lower temperatures also need to be simulated, such as those in the upper troposphere. In order to simulate atmospheric reactions at precisely controlled temperatures, different temperature control systems have been designed. For indoor smog chambers, the reactor is usually placed in a temperature-controlled room or an enclosure with an air conditioner. Meanwhile, the enclosure is usually lined with clear sheeting to maximize and homogenize the interior light intensity. For outdoor smog chambers, cooling the floor of the reactor, allowing the solar light to pass through the reactor, and using two layers of film can help to reduce the temperature increase due to absorption of solar irradiation.

### Monitoring system

The monitoring system is the most important part of a smog chamber system, since it determines how much we will know about the simulated atmospheric process. The monitoring system can be divided into three categories, i.e. meteorological conditions, gas phase and aerosol phase. The main instruments or technologies used in smog chamber experimental systems can be found in Table S3.

## DEVELOPMENT HISTORY OF SMOG CHAMBERS

### Smog chambers from the 1940s to 1960s

In the 1940s, the Los Angeles smog began to attract the attention of researchers. Haagensmit successfully simulated the formation of this pollution with the smog chamber technique, and revealed that the main source of secondary air pollutants such as O_3_ and organic peroxides was the photochemical reactions of VOCs and NO_x_ [36]. Smog chambers were also used to study the damage caused by photochemical smog on plants and the irritation to eyes, as well as the contribution of emission sources or chemical compounds to air pollution [37]. These investigations kicked off the study of atmospheric chemistry, with smog chambers simulating related atmospheric processes. During the 1950s and 1960s, smog chambers were usually retrofitted from green houses, which were mainly made from glass [37]. The concept of ‘twin reactors’ emerged during this period. The twin reactors allowed two experiments with almost identical conditions to be carried out simultaneously and had the advantage of identifying the influence of a single variable on the atmospheric process. This is crucial for ensuring that outdoor chambers can overcome the difficulty of reproducing weather conditions, such as temperature and irradiation.

### Smog chambers from the 1970s to 1980s

In the 1970s, researchers began to build smog chambers aimed at revealing the chemical processes in the atmospheric environment. Pitts *et al.* built a 6 m^3^ indoor smog chamber with an aluminum alloy coated with a fluorinated ethylene propylene (FEP) film [38]. This chamber could be evacuated and was equipped with artificial irradiation sources. After that, several smog chambers with volumes ranging from hundreds of liters to several cubic meters were built (Tables S1 and S2). These chambers were usually built from stainless steel or aluminum alloys coated with FEP film. Small-volume glass reactors were also used to study atmospheric reactions. Outdoor smog chambers were also developed in this period. Jeffries *et al.* [39] built two 156 m^3^ outdoor smog chambers with FEP film and studied photochemical reactions with solar irradiation. The transmittance of 280–460 nm light through the FEP film is >80%, which avoids the big difference in irradiation between the smog chamber and the real atmosphere. The functional testing and characterization of these indoor and outdoor smog chambers helped us understand the use of smog chambers for studying the atmospheric environment. Interference by the reactor wall was recognized.

Lots of experiments on the formation of photochemical smog and secondary inorganic aerosol, initially focused on sulfate, were carried out in these chambers with ambient air. Limited by the simple monitoring instruments available, the concentrations of pollutants were relatively high, and only some normal gas pollutants were measured, such as O_3_ and SO_2_. Meanwhile, due to the diversity of the ambient air used, the reproducibility of smog chamber experiments was not good. In order to minimize the interference of ambient air, purified air began to be used to clean the smog chambers in the 1980s. Adding specified pollutants into a smog chamber with a clean background allowed the study of specific atmospheric chemical reactions, such as O_3_ formation from the oxidation of VOCs. With the development of measurement technology of submicron particle numbers and sizes, smog chambers began to be used to investigate SOA formation, which became a research hotspot in post-1980s works [31].

### Smog chambers from the 1990s to 2000s

In the 1990s, more advanced smog chambers were built, and in greater numbers, including aerosol chambers and indoor and outdoor photochemical chambers, such as the 84 m^3^ Aerosol Interaction and Dynamics in the Atmosphere (AIDA) chamber built in 1990 [40,41], the two 200 m^3^ European Photoreactor (EUPHORE) outdoor chambers built in 1995 [42,43], the indoor 256 m^3^ chamber built in Jülich in 1996 [44] and the two 90 m^3^ indoor chambers built in Riverside in 2000 [45]. These chambers aimed to simulate atmospheric processes under conditions close to those in the real atmosphere, to obtain kinetic parameters, reaction mechanisms and yields of SOA and O_3_ from a single VOC, and further, to foster the development of air quality models. For example, the 256 m^3^ chamber built in Jülich was designed with a top that can be cooled and a bottom that can be heated. The temperature gradient then resulted in vertical mixing like that in the troposphere. This chamber was mainly used to study the NO_y_ chemical mechanisms at night-time. Based on the results obtained with these smog chambers, several atmospheric chemical mechanisms were developed, including the Carbon Bond Mechanism (CBM), Statewide Air Pollution Research Center Mechanism (SAPRC), Regional Acid Deposition Mechanism (RADM), Regional Atmospheric Chemical Mechanism (RACM), Master Chemical Mechanism (MCM) and Common Representative Intermediates (CRI). These mechanisms are still widely used in modern air quality models and play important roles in supporting the control of photochemical pollution.

In this period, wall effects, including absorption, deposition and heterogenous reactions of the reaction precursors, reactive intermediates and products on the wall, became further understood. The photolysis of HONO generated in the hydrolysis of NO_x_ on the reactor wall was recognized as an important OH radical source in the smog chamber. Wall losses were found to be underestimated in small smog chambers, and therefore resulted in significant underestimation of the yields of peroxides, O_3_ and secondary aerosols. Increasing the volume of chambers to reduce the wall effects became one obvious trend in chamber construction.

After 2000, newly built chambers aimed at achieving lower background effects and utilizing comprehensive monitoring technologies. The low background pollutant concentrations allowed simulation of the real atmosphere even in a very clean environment, meaning these more advanced chambers obtained more accurate information on atmospheric transformation. Meanwhile, the comprehensive monitoring systems had the ability to measure precursors, some key intermediates and radicals, and complex oxidation products in both the gas phase and aerosol phase on-line. Benefiting from these advanced monitoring technologies, researchers were able to simulate more complicated reaction systems, such as the photo-oxidation of emissions from mobile vehicles, biomass burning and plants rather than the photo-oxidation of a single VOC. More attention was also given to the effects of pollutants and their atmospheric oxidation products on health, climate and ecosystems. The two most representative chambers are SAPHIR and CLOUD, which were built in 2000 and 2006, respectively, and represented the current technical level. The details of some notable chambers and main research concerns are introduced here.

#### AIDA aerosol chamber

This chamber was made from aluminum (AlMg3), with a volume of 84 m^3^ [40]. It is a unique experimental facility in which experiments can be performed under atmospherically relevant conditions within a wide range of temperatures (−90–60°C), pressures (1 Pa–10^5^ Pa) and RH (0%–100%) [41]. It was mainly used to investigate aerosol formation and chemistry, and the direct and indirect effects of aerosols on climate, as well as the formation and characterization of the ice phase in clouds. Due to its well-controlled temperature and RH, AIDA can also be used for testing or calibrating related instruments.

#### EUPHORE outdoor chamber

EUPHORE had a wide involvement of institutes and advanced facility design, and was one of the most famous smog chambers in the world. EUPHORE has two FEP Teflon hemispherical reactors with a volume of 200 m^3^ each [42,43]. The semispherical shape of the reactor can be maintained when the pressure in the reactor is 100–200 Pa higher than the ambient air, or with the support of an epoxy resin tube in the reactor. The bottom of the reactor consists of 32 aluminum plates coated with FEP, which are connected to a cooling system to reduce the rise in temperature in the reactor due to the greenhouse effect. A window-shade cover was designed for each reactor to protect it from bad weather and adjust the irradiation intensity in the reactor. EUPHORE has mainly investigated the photochemical degradation of atmospheric pollutants and studied the generated products that present a potential risk to health and the environment. EUPHORE developed an atmospheric chemistry database with international projection, which caters for the development and validation of atmospheric chemical mechanisms such as MCM [42].

#### UCR indoor chamber

The UCR indoor chamber comprises two 90 m^3^ cuboid reactors made from FEP film [45]. These two reactors are in an enclosure in which the temperature (5–45°C) is well controlled by an air conditioner. Two kinds of light sources, including an Argon arc lamp and 80 black lights, are used to represent the spectrum of natural sunlight. A unique feature of the chamber is that the top of the reactor is equipped with an elevator system, which allows the top to move up and down. During experiments, the moveable top can adjust the volume of the reactor to compensate for sampling, leaks and permeation, maintaining a positive pressure in the reactor (5 Pa higher than the enclosure). The two reactors are connected through solenoid valves and blowers. The blowers and the Teflon-coated fans located within each reactor can mix the air in the two reactors and ensure that they have identical concentrations of mixed pollutants [45]. This chamber was mainly used to investigate the chemical pathways leading to SOA formation in the atmosphere and to study the impacts of emissions from diverse sources such as vehicles, wildfires, agricultural operations and consumer products, with the goal of developing new air quality models and improving the accuracy of existing ones, such as SAPRC.

#### SAPHIR outdoor chamber

SAPHIR, which is made from two layers of FEP, is the largest outdoor smog chamber, with a volume of 280–370 m^3^ [46]. The air between the two FEP layers is continuously flushed with purified air, which avoids pollution of the reactor due to leaks and permeation from the ambient air. The advantages of this chamber include its large volume, low background concentration, well-controlled temperature increase, adjustable irradiation intensity and advanced monitoring instruments such as laser induced fluorescence (LIF), differential optical absorption spectroscopy (DOAS) and matrix isolation electron spin resonance (MIESR) to measure HO_x_ and RO_2_ radicals. SAPHIR is used to investigate the key precursors and factors that determine the concentrations of radicals, the driving factors of photo-oxidant generation and degradation, the key parameters influencing the decay of trace pollutants and the physicochemical properties of aerosols. SAPHIR made serious improvements in the oxidation mechanism of semi-volatile organics and the formation of SOA, contributed to model development for ozonolysis and SOA formation chemistry, and did lots of work on evaluation of hygroscopic/optical properties and the health effects of SOA. Besides, with the help of SAPHIR, multiple monitoring technologies and instruments were developed, including those for the direct measurement of radicals (OH, HO_2_, RO_2_, NO_3_ and so on). Recently, a new plant chamber facility, PLant chamber Unit for Simulation (PLUS), was coupled to SAPHIR to investigate biogenic emissions and related atmospheric chemistry [47].

#### CLOUD indoor chamber

The CLOUD chamber is a 26 m^3^ stainless steel chamber built in the European Organization for Nuclear Research (CERN). The chamber was designed to achieve high-level cleanliness. The chamber is cleaned with dry high-pressure ultrapure air at room temperature, wet ultrapure air at high temperature (373 K) and ultrapure air with a ppm level of O_3_. The nucleating agents in CLOUD are controlled at realistic concentration levels (ppq level) [48,49]. Organic components are not used in the sampling or air supplying lines. The chamber is placed in an insulated thermal housing, which can adjust the temperature of the chamber between 207 K to 310 K with high stability. Two magnetically driven stainless steel fans are used to mix the air in the chamber [50]. The RH in the CLOUD chamber is controlled by introducing humid air, which is heated in the tube to avoid water condensation. Irradiation with wavelengths in the range 250–750 nm is produced by four Hamamatsu LC8 UV lights (200W Hg-Xe lamp) and introduced via 239 optical fiber vacuum feedthroughs [51]. The UV light intensity in the chamber is adjustable (0 to 124 mW m^–2^), which can adjust the H_2_SO_4_ production rate in the chamber. The most unique feature of the chamber is that the ion concentrations in the chamber can be adjusted. The chamber can be exposed to either galactic cosmic rays or a 3.5 GeV c^–1^ secondary pion beam (π beam) from the CERN Proton Synchrotron. The beam intensity can be adjusted from 200 to 600 ion-pair cm^–3^, corresponding to the natural ion-pair concentration range from ground level to stratospheric values [52]. A high-voltage clearing electric field (up to 20 kV m^–1^) can be generated inside the chamber, which can reduce the ion-pair concentration to a few ion-pair cm^–3^ within a second. State-of-the-art instruments are available for use with the chamber during the experiments, including various chemical-ionization mass spectrometer (CIMS) and particle sizers to measure ions, particles and trace gases.

### Smog chambers in China

Smog chamber research in China started relatively late. In the 1980s, Tang *et al.* [53] in Peking University established the earliest indoor photochemical smog chamber, which mainly focused on studying the photochemical smog in Lanzhou, China. Since then, a series of smog chambers with volumes of several hundred liters to several cubic meters were successively built to study gas-phase kinetic mechanisms and SOA formation (Tables S1 and S2). However, these small-volume smog chambers have the disadvantage of relatively large wall effects, and it is also difficult to conduct long duration experiments. In recent years, many smog chambers with volumes over 10 m^3^ have been developed around China to study secondary aerosol and O_3_ formation under various conditions, such as the 30 m^3^ indoor smog chambers at the Guangzhou Institute of Geochemistry, Chinese Academy of Sciences (GIG-CAS) [54] and RCEES-CAS [23], and the 45 m^3^ outdoor smog chamber in the Chinese Research Academy of Environmental Sciences (CRAES) [55]. In order to control PM_2.5_ and O_3_ pollution in China, the development of smog chambers has become imperative to deeply understand the complex air pollution, especially in China's megacities. A total of 35 research institutes and groups in China have built smog chamber systems, of which indoor smog chambers are the main ones (http://www.bjkwnt.com/ditu/index.html). Compared with smog chambers in developed countries, the volume of chambers in China is still relatively small. Meanwhile, since one chamber is usually operated by a single small research group, the equipped instrumentation is relatively lacking, especially for the detection of radicals and intermediate products. Therefore, more high-quality smog chambers with larger volumes, equipped with state-of-the-art instruments, are urgently needed in China.

## MAIN RESEARCH TOPICS AND ACHIEVEMENTS

With the development of smog chamber technology, atmospheric chemistry saw great progress. Since many review articles and books have summarized these achievements [31,56–60], here we list some representative achievements in which smog chambers made important contributions.

### O_3_ chemistry

**Figure 2.**
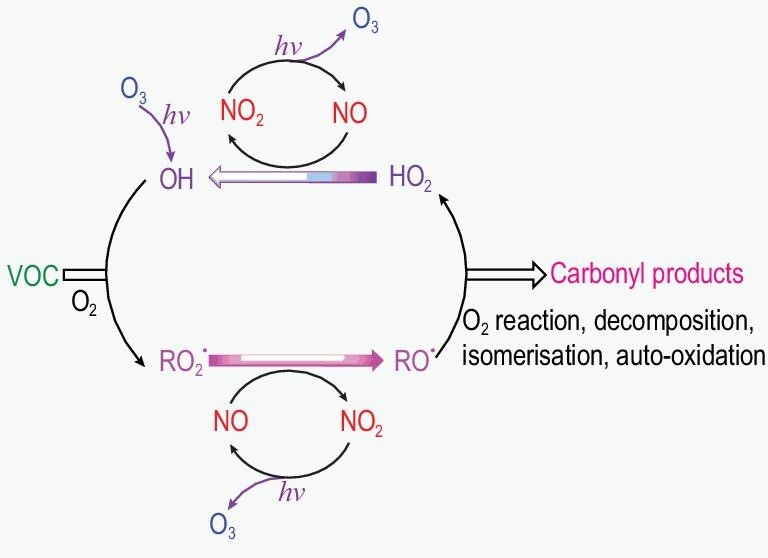
NO_x_-HO_x_-RO_x_ cycles in atmospheric photochemical reactions.

Ever since the famous flask experiments [61], O_3_ generation has been confirmed in photochemical smog. The NO-NO_2_-O_3_ redox cycle (Chapman cycle, Fig. 2)—i.e. NO_2_ is photolyzed into NO under irradiation and NO reacts with O_3_ to return to NO_2_—will not generate a net gain of O_3_. In the presence of VOCs, however, NO is oxidized to NO_2_ not only by O_3_ but also by other photo-oxidants, such as RO_2_ radicals, which are generated from reactions between the VOCs and HO_x_ radicals. The additional conversion of NO to NO_2_ will then result in the accumulation of O_3_. In turn, O_3_ photolysis is an important source of OH radical and promotes the oxidation of VOCs, which results in the formation of other secondary pollutants (e.g. peroxylacyl nitrates (PAN) and SOA). The coupled NO_x_-HO_x_-RO_x_ cycle was revealed as the key reaction scheme in atmospheric photochemical reactions. The massive coexistence of NO_x_ and VOCs was revealed as the main cause of O_3_ pollution in Los Angeles smog, and these pollutants were mainly derived from vehicular emissions and industrial fumes. These scientific findings supported the control of NO_x_ and VOCs emissions from transportation and industry in order to reduce photochemical pollution in the city.

To this day, O_3_ pollution is still a problem in many cities in both developing countries and developed countries. The broad array of sources of the precursors and the nonlinear response of O_3_ to its precursors provide a significant challenge to O_3_ pollution control. As VOCs and NO_x_ were recognized as the two most important precursors for O_3_ formation, their relative abundance was further known to be important for determining O_3_ concentrations [24]. The sensitivity of O_3_ formation to VOCs or NO_x_ concentrations was calculated using the empirical kinetic modeling approach (EKMA) chemical model, which then provided the basic scientific support for the control of O_3_ pollution. As there are hundreds of VOCs in the atmosphere, it is crucial to know which VOCs were the most important precursors. The O_3_ formation potential of single VOCs has been widely investigated in smog chambers to evaluate the contribution of VOC emission to O_3_ formation. Alternatively, the incremental reactivity to yield O_3_ formation can also be tested by adding a VOC to the mixture and recording the change in O_3_ production. Knowledge of the contribution of speciated VOCs or emission sources based on smog chamber experiments was the precondition of an accurate control of O_3_ pollution.

### VOC oxidation

**Figure 3.**
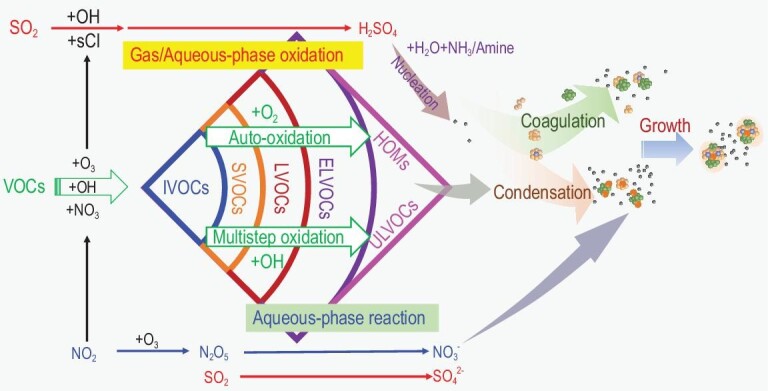
Precursor oxidation, aerosol formation and growth.

Large quantities of VOCs (including alkenes, alkanes, aromatics and oxygenates) are emitted into the troposphere from anthropogenic and biogenic sources. Understanding their chemical behaviors is crucial to predicting the atmospheric environment. Gas-phase oxidation mechanisms of VOCs initiated by OH, O_3_, NO_3_, etc. have been developed over the past 70 years (Fig. 3). For the majority of these, reaction with OH is the dominant or exclusive removal process, which plays an important role in determining the atmospheric lifetime of the VOC. The formation of alkyl radicals (R), alkoxyl radicals (RO), RO_2_ and Criegee intermediates (CI) [62,63], as well as the generation of oxidation products with different volatility (e.g. intermediate volatility organic compounds (IVOCs), semi-volatile organic compounds (SVOCs), low-volatility organic compounds (LVOCs), extremely low volatility organic compounds (ELVOCs) and ultra-low volatility organic compounds (ULVOCs)) in the subsequent reactions, such as auto-oxidation [64–66], reacting with NO_x_, and polymerization reactions, were recognized as the main transformation paths of VOCs in atmospheric oxidation. With the help of smog chamber experiments, oxidation mechanisms of VOCs, with consideration of the key factors influencing oxidation, such as NO_x_ level [67], were revealed. Meanwhile, VOC oxidation kinetics were widely studied under atmosphere-relevant conditions. These kinetics, which were summarized in previous review articles [68,69], are the basis of air quality models used to predict O_3_ and SOA formation.

### NPF and gas-particle partitioning

NPF is an important source of cloud condensation nuclei and has been subjected to intensive study. The picture of NPF and growth is not fuzzy anymore with the aid of chamber simulation, despite the fact that nucleation mechanisms in various atmospheric environments are still to be elucidated, which hampers the understanding of the aerosol effect on climate. Studies based on CLOUD chamber have reported the nucleation mechanism from sulfuric acid-water-ammonium, sulfuric acid-water-ammine, sulfuric acid-organic vapor-water, pure organics, and iodic acid [48,70–72]. With different experimental conditions representing the atmosphere in different continental and marine environments, the experimental results reveal several corresponding nucleation mechanisms. For example, inspired by the fast nucleation rate from CLOUD chamber experiments, Yao *et al.* [73] reveal that nucleation from sulfuric-dimethylamine-water is a dominant NPF mechanism in urban Shanghai. However, Cai *et al.* [74] pointed out that typical conditions in Beijing and Nanjing are different from those used in CLOUD experiments. Gaseous precursors and condensation sink, at their typical ambient levels, are needed for future chamber studies to better represent polluted conditions. In addition, with the presence of ions, which are generated due to the simulated galactic cosmic ray in the chamber, the nucleation rate is remarkably enhanced compared with the condition without ions [48,71]. These findings have greatly advanced the understanding of the NPF mechanism and its effect on air quality and climate.

After nucleation, the growth of these newly formed particles is governed by the condensation of low volatility vapors, which is also called gas-particle partitioning [75,76]. The condensation of H_2_SO_4_ and its clusters leads to the enhanced growth rate of newly formed particles [77,78]. In addition, chamber experiments conducted in the Julich Plant Atmosphere Chamber showed that highly oxygenated organic molecules (HOMs) with low volatility can also contribute to the growth of newly formed particles [64]. Wang *et al.* [79] showed that the condensation of nitric acid and ammonia contribute to the rapid growth of newly formed particles in CLOUD chamber experiments, which could explain the high survival rate of newly formed particles in urban environments, though direct atmospheric evidence is needed to confirm its relevance in the real atmosphere.

### Aerosol chemistry

A large amount of secondary aerosol mass was also found to be generated, accompanied by the oxidation of gas precursors, which was verified in laboratory experiments [80]. The oxidation of SO_2_ in photochemical reactions will form sulfuric acid and sulfate, while the low-volatility organic products produced from the oxidation of VOCs will partition into the aerosol phase and result in SOA formation. For the nitrate formation, the oxidation of NO_2_ by OH during the day and the hydrolysis of N_2_O_5_ at night are the dominant reaction pathways. Sulfate, nitrate and SOA are important compositions of atmospheric fine particles and are still research hotspots today. It was found that hydrocarbons and oxygenates with a carbon number of >2 would have the potential to contribute to SOA formation [81]. Smog chambers are the main way to quantify the SOA formation potential of single VOCs. In order to do this, multiple methods have been used, including ‘fractional aerosol coefficient’ (FAC) [82], SOA yield [83] and volatility basis set (VBS) [84]. FAC is a simple ratio, which estimates SOA formation from one VOC by multiplying its ‘initial concentration’ in the chamber (emission) by its FAC. SOA yield is a better method than FAC, which is not a unique value but rather a function of the available absorbing organic aerosol concentration [85]. To characterize this dependence, one-product or two-product models were applied to smog chamber studies. In these models, the yield of condensable oxidation products and their ability to partition to the aerosol phase, which mainly depend on volatility, were both considered. With better understanding of the oxidation mechanisms and the development of more advanced analysis technology, researchers gained more and more information on the organic products of the oxidation of VOCs, and were able to characterize the SOA yield with more elaborate approaches, such as VBS. VBS is a uniform basis set of saturation vapor pressures spanning the range of ambient organic saturation concentrations, where chemical evolution can be treated by a transformation matrix coupling the various basis vectors [84]. Besides gas-phase oxidation, chemical evolution in the aerosol phase was also found to have important impacts on SOA formation. One example is that acid seed particles were found to enhance SOA formation through acid-catalysis reactions [86,87]. These acid-catalysis reactions will contribute significantly to atmospheric aerosols in the presence of high concentrations of sulfate or SO_2_ [6]. Chambers for cloud and aerosol studies were also used to investigate the role of aqueous aerosol chemistry in aerosol formation, which found that key steps of chemical conversions occur within water-containing aerosol particles, and have become a complementary tool for bulk-phase studies. This significant progress has been summarized in a previous review [88] and in references therein.

### Supporting numerical simulation and air pollution control

One major contribution of smog chamber data is to verify and evaluate atmosphere-related mechanisms. Detailed gas-phase mechanisms involving more than 20 000 reaction pathways and thousands of trace species have been constructed since the 1950s [89]. Jenkin *et al.* [90] and Saunders *et al.* [91] developed a near-explicit MCM to describe the detailed gas-phase chemical processes involving NO_y_ and a series of primary emitted VOCs. Meanwhile, the smog chamber dataset was used to update and evaluate the MCM mechanisms to constantly improve the gap between model and measurement [42]. Other condensed mechanisms such as CBM [92], RADM [93] and the SAPRC series [94] were also developed and have evolved since their first versions. Besides the evaluation of chemical kinetics, smog chambers have been applied for instrument intercomparisons and interferences [95,96]. The development of advanced monitoring technologies in turn strengthens the ability of a smog chamber to explore atmospheric processes. The revealed new reaction pathways for precursor oxidation, new mechanisms of nucleation, particle growth and aerosol aging in smog chamber simulations are being added into different models periodically. For example, the reported heterogeneous oxidation of SO_2_ in the presence of NO_2_ and NH_3_ helped to improve sulfate simulation in heavy haze [97]. Some findings in smog chambers helped researchers to understand the effects of aerosol on climate. For example, with the advanced understanding of the aerosol formation mechanism in the CLOUD chamber, Dunne *et al.* [98] constructed a model and found that cosmic ray intensity cannot meaningfully affect climate via nucleation in the present-day atmosphere.

There are also a few limitations when applying smog chamber results to air quality models. A smog chamber has the advantage of simulating gas-phase reactions, while it is usually difficult to explore microscopic heterogeneous chemical processes due to the difficulty of applying *insitu* surface analysis technologies to suspended aerosols in the chamber. The interferences from the chamber wall, which usually lead to uncertainty in smog chamber results, always exist and should be carefully considered. These interferences, and the attempt to better represent real atmospheric environmental conditions, also result in a relatively complex reaction system in the chamber, and therefore it is usually not easy to quantify the contribution of a specific reaction pathway. Besides, due to wall losses, it is difficult for smog chambers to simulate atmospheric aging processes over long residence times (such as multiple days). Numerical simulation and smog chamber simulation have to work together to explore the complex processes in the atmospheric environment.

The knowledge of atmosphere-related mechanisms and the further development of air quality models provided crucial support for the proposal of regulatory measures, and contributed scientific support for air pollution control. For example, smog chamber simulation of the photochemical reactions of NO_x_ and VOCs initiated a cooperative research entity with industrial or motor vehicle representatives and led to the creation of the Air Pollution Foundation in 1954 [31], and guided the control of VOCs and NO_x_ to reduce O_3_ pollution in California. These studies on the Los Angeles smog caused by the photoreaction of NO_x_ and VOCs gave birth to the famous ‘Clean Air Act’ in the United States, administered by the US Environmental Protection Agency (EPA). This is one of the first and most influential modern environmental laws in the US, as well as one of the most comprehensive air quality laws in the world. This law promoted the development of vehicle exhaust pollution control technology. This also in turn promoted the development of major smog chamber facilities around the world to ultimately deal with the air quality. Meanwhile, the Community Multiscale Air Quality (CMAQ) model developed by the US EPA during this period has also become a universal and mainstream air quality model worldwide.

## KEY FACTORS INFLUENCING CHAMBER SIMULATIONS

In order to obtain a high-fidelity simulation of accurate atmospheric chemistry and kinetics, smog chambers are expected to have sufficiently low background pollutant levels, low wall reactivity and wall loss, good simulation of solar irradiation, well-controlled temperature and RH, and so on. Some of these factors and their influence on the simulation of the atmospheric environment are discussed here.

### Background pollutant levels

The background pollutant level can be minimized by purifying the background air and taking steps to reduce the introduction of ambient pollutants due to leaks or permeation. Non-reactive tracer compound, such as Hexafluorobenzene (C_6_F_6_), sulfur hexafluoride (SF_6_) or acetonitrile (CH_3_CN), can be introduced for leak detection. FEP Teflon, which is the most widely used material for smog chambers to date, is known to allow the permeation of water molecules, and can cause leaks when the welding of FEP Teflon pieces is not tight enough. Different thicknesses of FEP Teflon (50–250 μm) have been used for different smog chambers (Tables S1 and S2). The thickness of the FEP Teflon will influence the permeation, weld and subsequent leaks in the chamber, while these differences have not been comprehensively studied as far as we know. Before a smog chamber experiment, a continuous flow of clean air is usually introduced into the chamber for cleaning purposes. Besides flushing, heating the reactor, introducing high O_3_ concentrations and UV irradiation were also used to remove the residual organics in the chamber. Further photochemical cleaning was also performed by using H_2_O_2_ and NO in some chambers [99]. To study pure neutral nucleation, a high voltage can be introduced to generate an electric field gradient to remove the ions in a chamber [48,49].

Contamination in chambers has been discussed as a concern since the 1970s. The pollutant contamination levels should be controlled to below the detection limit of the instruments in general, but of course this is case-dependent. There is always a higher tolerance for inert pollutants than active pollutants, and the tolerance is highly dependent on the purpose of the simulation. For example, the CO_2_ concentration is usually higher than the ppm level in chambers, while the concentrations of nucleating agents such as H_2_SO_4_ can be controlled at the ppq level in the CLOUD chamber [48,49]. Background pollutant levels in some smog chambers are listed in Table S4.

### Wall loss

The chamber wall causes the main uncertainty in smog chamber data [57]; the loss of gaseous pollutants, radicals and particles on the wall plays an important role [100]. The deposition rates of these species are influenced by the reactor volume and shape, the wall material and the mixing inside the reactor. The deposition rate is also temperature- and species-dependent. For gas pollutants, the deposition rates of NO_x_ and O_3_ in smog chambers have been widely reported, and are usually in the range 10^–5^–10^–4^ min^–1^. Hydrocarbons were reported to deposit at slightly higher rates [101] or slower rates [54] than the deposition of NO_2_ and O_3_ in a given chamber. The wall loss rates of pollutants are summarized in Table S5.

The wall loss of radicals (e.g. OH radicals) will greatly affect the formation of secondary pollutants (e.g. O_3_ and SOA). For example, the loss of OH radicals to the wall or aerosols will be enhanced by elevated RH [102], which will further influence the gas-phase oxidation capacity of reaction systems, as well as the O_3_ and SOA formation. Due to the extremely high occurrence of molecular collision, it can be speculated that the loss of radicals is also greatly affected by the volume of the smog chamber due to their extremely high activity. Chambers with larger volume would have relatively lower wall loss of radicals. Organic vapor and particle loss on Teflon surfaces will cause an underestimation of particle yield [103]. Quantifying the deposition loss of SVOCs and LVOCs, which is assumed to be reversible, is more difficult than for NO_x_ and O_3_. Considering that real-time detection instruments for low-volatility or semi-volatile organics are not widely available, it is difficult to quantify or even identify their wall losses experimentally, but they can be accounted for empirically. Wall loss of SVOCs and LVOCs depends on the equivalent wall OA concentration (*C*_w_) and their vapor saturation concentrations (*C*_i_^*^) [103,104]. The overall vapor wall loss rate coefficient (*k*_w_) is dependent on the S/V ratio of the chamber, the degree of turbulent mixing in the chamber, the molecular diffusivity of the vapor and the mass-accommodation coefficient [103,105,106]. The detailed relationship between *k*_w_ and the S/V ratio can be expressed as follows:

$$\begin{eqnarray*}
{\rm{\ }}{{{k}}_{\rm{w}}} = \frac{{\rm{S}}}{{\rm{V}}}{\rm{\ }} \times \frac{{\frac{{{{\rm{\alpha }}_{\rm{w}}}{\rm{\bar{c}}}}}{4}}}{{1.0 + \frac{{\rm{\pi }}}{2} \times \left[ {\frac{{{{\rm{\alpha }}_{{\rm w}}}{\rm{\bar{c}}}}}{{4{{\left( {{{{k}}_{\rm{e}}}{{{D}}_{{\rm{gas}}}}} \right)}^{0.5}}}}} \right]}},
\end{eqnarray*}$$

in which S and V are the surface and volume of the smog chamber, respectively. α_w_ is the mass-accommodation coefficient of vapors onto the chamber walls, ${\rm{\bar{c}}}$ is the mean thermal speed of the molecules, *k*_e_ is the coefficient of eddy diffusion and *D*_gas_ is the gas-phase diffusivity, which is assumed to vary with molecular weight (MW) and is equal to ${{{D}}_{{\rm{C}}{{\rm{O}}_2}}}$(${\rm{M}}{{\rm{W}}_{{\rm{C}}{{\rm{O}}_2}}}/{\rm{MW}}$), with ${{{D}}_{{\rm{C}}{{\rm{O}}_2}}}$= 1.38 × 10^–5^ m^2^ s^–1^.

For a given vapor molecule, the mean thermal speed ${\rm{\bar{c}}}$ could be calculated according to the following equation:

$${\rm{\bar{c}}} = \sqrt {\frac{{8{\rm{RT}}}}{{{\rm{\pi MW}}}}},]$$

in which R is the ideal gas constant (i.e. 8.314 J mol^–1^ K^–1^), T is the experimental temperature, and MW is the molecular weight.

The deposition rate of aerosol is size-dependent, and a series of methods have been developed to correct aerosol wall loss according to the available instruments. According to the test results in some experiments, aerosols with a diameter of ∼300 nm usually deposit slowest, while smaller aerosols and larger particles deposit faster due to diffusion and gravity deposition, respectively [25]. Some previous studies reported the lifetimes of aerosols without providing the range of aerosol diameters. Testing the loss of particles over the widest range that instruments can cover is strongly recommended for characterization of chambers. In any case, the lifetimes of aerosols are quite different in different smog chambers with different volumes and different wall materials. Aerosol deposition is faster in smaller chambers due to the fact that they have a higher S/V ratio than larger chambers. Meanwhile, due to static electricity, aerosol deposition on Teflon walls is generally quicker than that on metal walls. Aerosol deposition rates or lifetimes in some chambers are also listed in Table S5. The charge of particles or ions also had a great effect on their deposition in smog chambers [26]. Grounded stainless steel was also found to be good at controlling the concentration of ions, while in a Teflon chamber most ions would be stripped away, and charges accumulating on the Teflon surfaces produce uncontrollable electric fields [49].

For a better application of smog chambers, a series of comprehensive computational models were also developed to understand the complex physicochemical processes associated with the deposition of vapors on the wall and on suspended aerosols, deposition of aerosols on the wall, and coagulation and condensation of suspended aerosols [106–108].

### Wall emission

Besides acting as deposition sinks for pollutants, walls can also act as emission sources. The pollutants deposited on the wall can partition back to the gas phase when the gas wall equilibrium changes. For example, the emission of NH_3_ from deposited ammonium sulfate was reported to be the main reason for background NH_3_ [6]. More importantly, heterogeneous reactions could occur on the walls and have significant impacts on the chemical processes in the chamber. For example, the heterogeneous reaction of NO_2_ on the wall has been recognized as a HONO source for a long time. The photolysis of HONO will then provide OH radical and initiate the oxidation of VOCs and many other pollutants. The uptake of NO_2_ and HONO formation rates showed orders of magnitude differences in different chambers. For example, under irradiation conditions, the uptake constant of NO_2_ was reported to be as high as 10^–2^ s^–1^ with a HONO yield of 10% in a 4.2 m^3^ stainless steel chamber [109], while it was <10^–5^ s^–1^ in large Teflon chambers (24 and 27 m^3^) [101,110]. The HONO formation rate could be influenced by the materials of the chamber walls [111], and was proportional to the surface area in the chamber [112]. Smog chamber walls served as a stronger HONO source under irradiation than under dark conditions [110], and under humid conditions than under dry conditions [113]. The photolytic HONO source was found to be proportional to the photolysis frequency of NO_2_ [112]. With irradiation, the HONO emission rate from chamber walls could be as high as 10^5^–10^7^ molecules cm^–3^ s^–1^ [110,114,115]. In addition, the photolytic HONO source increased with the square of RH [46], and the photo-enhanced reaction of NO_2_ and water was postulated to be responsible for the formation of HONO [116]. The photolytic HONO source also increased exponentially with temperature [46]. Besides the heterogeneous reactions of NO_2_, the photolysis of nitrate adsorbed on the surface was also postulated as a HONO source [111], while the photolysis of nitric acid on the chamber wall can also serve as an OH radical source directly [110].

## NEXT-GENERATION SMOG CHAMBERS

Smog chambers have been built worldwide and will continuously make significant contributions to the study of the atmospheric environment and the improvement of air quality. With more than 70 years’ development, current smog chambers are already successful in some specific investigation directions, although the technologies can always be further improved. Despite the progress in atmospheric chemistry related to smog chambers, there are also emerging problems in our atmospheric environment that the developed mechanisms could not fully explain, such as the temperature increase in outdoor chambers due to the greenhouse effect, underestimation of wall effects, limited knowledge about the chemical composition of the generated aerosols, and the difficulty in detecting intermediate compounds, such as radicals and clusters. No heavily invested large-volume chambers have been built since 2010. For next-generation smog chambers, more diversified simulation would be expected with more advanced and more comprehensive simulation technologies, and the following development directions may need to be considered.

### High-fidelity simulation of atmospheric photochemistry

#### Large volume

To simulate atmospheric chemical processes under conditions as close as possible to those in the real atmosphere, a reactor with a large volume is needed to minimize wall effects, including both deposition and heterogeneous reaction on the wall. The large volume will also help in longer-duration simulation, for example, longer than one day, which is important for studying the atmospheric aging process. A well-designed mixing system should be introduced to ensure homogeneity in the chamber and to overcome the potential accumulation of air stratification, as summarized by Hidy [31].

#### High cleanliness

The background pollutant concentrations should be controlled at minimal levels. The influence of the background pollutants on the investigated reaction system should be well characterized.

#### Well-controlled reaction conditions

Experimental conditions should be controlled as much as possible to conform to those in the real atmosphere. The pollutant reactants as well as secondary reaction products should be constrained within concentration ranges in the real atmosphere. One should try to use ambient irradiation or at least well-characterized artificial irradiation sources, or both. The temperature, RH, and air pressure in the chamber should be well-documented.

### Advanced detection system

The ability to accurately detect relevant species is crucial for revealing atmospheric processes. An advanced monitoring system, including detection of gases, radicals, ions and particles, should be developed according to the properties of the chamber facility and the experimental purposes. The chamber should be designed to be compatible with the fast development of instruments.

### Multi-chamber combo

As pointed out by Hidy [31], smog chambers are limited representations of the atmosphere. To understand complicated atmospheric translation processes, the combined effects of precursor emissions, and surface and multi-media exchange, hydrometeor interactions should be addressed. Besides, the interactions between these atmospheric processes with the changing climate in the context of carbon neutrality need to be urgently explored. A multi-chamber combo may be helpful in this regard. A combination of photochemical chamber, aerosol chamber and exposure chambers for health and ecological effects are expected to give new insight into complicated secondary aerosol formation processes, as well as their impact on climate, human health and the ecosystem. A multi-chamber combo may also serve to study cross-media environment problems, and help to predict how the Earth climate and surface environment will change in the future.

### A closed chain with field observation and numerical simulation

As mentioned earlier, field observation, laboratory study and numerical simulation are the three main approaches to investigating the physical and chemical processes in the atmosphere and their impacts on our environment, with both advantages and disadvantages for each. Chamber study should work closely with field observation and numerical simulation. The experimental conditions should always be comparable to those observed in the field, and the experimental results should be validated as much as possible with field observations. Applying smog chamber results in numerical simulation is the most common method to evaluate the importance of a laboratory finding. Therefore, with input from observations, simulation in smog chambers serves the development of air quality models, which in turn will be validated by observations, forming a closed chain for studying the atmospheric environment. In addition, it is highly recommended that a ‘numerical chamber’ associated with the smog chamber is developed, with knowledge of the wall effects, background impact and possible dilution and leakage considered. Comparisons between the mechanistic calculations and smog chamber measurements will provide detailed evaluation of chemical mechanisms and should be quite helpful in identifying accurate mechanisms and kinetics from smog chamber simulations.

The next-generation smog chamber, with more advanced simulation technology, more comprehensive functions and closer cooperation with observation and numerical simulation, will certainly prove its mettle in supporting the continuous improvement of air quality in the world.

## Supplementary Material

nwab103_Supplemental_File
