# Supplementary material for: Application of smog chambers in atmospheric process studies
Source: Natl Sci Rev. 2021 Jun 15;9(2):nwab103. doi: 10.1093/nsr/nwab103 (PMC8826053; doi:10.1093/nsr/nwab103)
Supplement: nwab103_Supplemental_File [file nwab103_supplemental_file.docx]

Supplementary Materials for

**Application of Smog Chambers in Atmospheric Process Studies**

Biwu Chu^1, 2, 3, #^, Tianzeng Chen^1, #^, Yongchun Liu^4^, Qingxin Ma^1, 2, 3^, Yujing Mu^1, 2, 3^, Yonghong Wang^1^, Jinzhu Ma^1, 2, 3^, Peng Zhang^1^, Jun Liu^1, 3^, Chunshan Liu^5^, Huaqiao Gui^6^, Renzhi Hu^6^, Bo Hu^7^, Xinming Wang^2, 3, 8^, Yuesi Wang^2, 7^, Jianguo Liu^2, 3, 6^, Pinhua Xie^2, 3, 6^, Jianmin Chen^2, 9^, Qian Liu^3, 10^, Jingkun Jiang^11^, Junhua Li^11^, Kebin He^2, 11^, Wenqing Liu^2, 3, 6^, Guibin Jiang^3, 10^, Jiming Hao^11^, Hong He^1, 2, 3, *^

^1^ State Key Joint Laboratory of Environment Simulation and Pollution Control, Research Center for Eco-Environmental Sciences, Chinese Academy of Sciences, Beijing 100085, China

^2^ Center for Excellence in Regional Atmospheric Environment, Institute of Urban Environment, Chinese Academy of Sciences, Xiamen 361021, China

^3^ University of Chinese Academy of Sciences, Beijing 100049, China

^4^ Aerosol and Haze Laboratory, Beijing Advanced Innovation Center for Soft Matter Science and Engineering, Beijing University of Chemical Technology, Beijing, China

^5^ Beijing Convenient Environmental Tech Co. Ltd, Beijing 101115, China

^6^ Key Laboratory of Environmental Optics and Technology, Anhui Institutes of Optics and Fine Mechanics, Chinese Academy of Sciences, Hefei 230031, China

^7^ State Key Laboratory of Atmospheric Boundary Layer Physics and Atmospheric Chemistry, Institute of Atmospheric Physics, Chinese Academy of Sciences, Beijing 100029, China

^8^ State Key Laboratory of Organic Geochemistry and Guangdong Provincial Key Laboratory of Environmental Protection and Resources Utilization, Guangzhou Institute of Geochemistry, Chinese Academy of Sciences, Guangzhou, 510640, China

^9^ Shanghai Key Laboratory of Atmospheric Particle Pollution and Prevention, Department of Environmental Science and Engineering, Fudan University, Shanghai, 200438, China

^10^ State Key Laboratory of Environmental Chemistry and Ecotoxicology, Research Center for Eco-Environmental Sciences, Chinese Academy of Sciences, Beijing 100085, China

^11^ State Key Joint Laboratory of Environment Simulation and Pollution Control, School of Environment, Tsinghua University, 100084 Beijing, China

^#^ These authors contributed equally to this work.

^*^ Corresponding author: honghe@rcees.ac.cn (Hong He)

#### Table S1. The typical indoor smog chambers around the world

| Year* | Chamber/Institute/Place | Volume (m^3^) | S/V  (m^-1^) | Material | Light sources | *J*_NO2_ (min^-1^) | Reference | Note (Features/Website) |
| --- | --- | --- | --- | --- | --- | --- | --- | --- |
| 1950s | Midwest Research Institute | ~62 |  | Greenhouse with glass |  |  | (1) |  |
| 1975 | Dearborn |  |  | Pyrex bell jar | Black light fluorescence lamps |  | (2) |  |
| 1979 | Riverside | 6.4 |  | All-glass (Pyrex) | Black light lamps |  | (3) |  |
| 1977 | EC Chamber, Riverside | 5.8 |  | Aluminum alloy coated with Teflon |  |  | (4-6) | Under vacuum, temperature controlled |
| 1982-1995 | ITC/ETC/DTC/XTC/CTC, Riverside | 3.5-6 |  | Teflon |  |  | (7) (8) |  |
| 1978 | TVA, Riverside | 28 |  | Teflon | 3 kinds of lights (280~ 450 nm) |  | (9) |  |
| 1979 | Tsukuba | 6.065 |  | Stainless steel lined with PFA | Xenon arc lamp |  | (10) |  |
| 1986 | Wuppertal | 1.08 |  | Quartz |  |  | (11) | Under vacuum, temperature controlled (0-25℃) |
| 1986 | Australia | 4×0.2 |  | FEP Teflon |  |  | (12) |  |
| 1990 | AIDA, KIT, Karlsruhe | 84 | 1.2 | Aluminum (ALMg3) | for dark experiments only |  | (13)  (14)  (15) | Temperature (+50°C to -90°C), pressure (1 to 1000 hPa), RH (0% to 100%); N_2_O_5_ loss rate: 8×10^-4^ s^-1^  <http://www.imk-aaf.kit.edu/73.php> |
| 1992 | EPA | 9 |  | Teflon |  |  | (16) | Indoor and mobile chamber |
| 2005 | EPA | 14.5 |  | Stainless steel with TFE Teflon | UVA-340 bulbs and standard UV bulbs | 0.34 | (17) |  |
| 1996 | Jülich | 256 |  | FEP Teflon |  |  | (18) | Dark experiments |
| 1997 | EC chamber, Toronto | 9 | 2.7 | Teflon | Phillips fluorescent UV-A & Sylvania black light lamps |  | (19, 20) |  |
| 1998 | Pasadena | 2×28 |  | Teflon |  |  | (21) | Temperature (10-40°C) |
| 2000 | UCR, Riverside | 2×90 |  | 125 μm FEP Teflon | 200 kW Argon arc lamp & 115W Black light lamps |  | (22) | Temperature (5-45°C) |
| 2003 | TCRDL, Japan | 2 |  | Teflon |  |  | (23) |  |
| 2003 | KIST | 2.5 |  | FEP Teflon |  | 1.10 | (24) |  |
| 2004 | PSI Chamber | 27 | 2 | FEP Teflon | 4 xenon lamps & 80 black light lamps | 0.12 | (25)  (26) | Temperature (17-25℃); <https://www.psi.ch/de/lac/smog-chamber> |
| 2013 | Mobile Smog Chamber, PSI | 9 | 3.2 | Teflon | 40 100W UV lights | 0.18 | (27) | There is also a “Cool chamber” (-10 to 30 °C; 0-95%RH) |
| 2005 | CSIRO | 18.1 | 2.36 | FEP Teflon | 40 blacklight  tubes (36W Sylvania Blacklight Blue 350) | 0.61-0.86 | (28) |  |
| 2018 | CSIRO | 24.8 |  | FEP Teflon | NEC FL40SBL (350 nm blacklight) & Sylvania F36W BLB (blue blacklight) | 0.44-0.52 | (29) |  |
| 2006 | CLOUD | 26 |  | Stainless steel | four Hamamatsu LC8 UV light sources (200W Hg-Xe lamp) |  | (30, 31) |  |
| 2007 | Carnegie Mellon Univ. | 10 |  | Teflon (PTFE/FEP) | Black lights |  | (32)  (33) | 288–313K |
| 2007 | HIRAC, University of Leeds | 2 | 5.8 | Stainless steel | Philips, TL-D36W/BLB | 0.12 | (34) | **Temperature range:** 250-350 K  **Pressure range:** 50-1000 mbar  <https://hirac.leeds.ac.uk> |
| 2007 | University College Cork Ireland | 6.5 |  | FEP | Black lights |  | (35)  (36) |  |
| 2007 | Leipziger Aerosolkammer (LEAK) | 19.0 | 2.0 | Teflon FEP | Fifty-six UV lamps (100W Eversun Super, Phillips) | 0.384 | (37, 38) | [http://www.tropos.de/en/research/projects-infrastructures-technology/technology-at-tropos](http://www.tropos.de/en/research/projects-infrastructures-technology/technology-at-tropos/aerosol-research-facilities/aerosol-chamber-experiments/) |
| 2009 | HEC Chamber, Harvard | 4.7 |  | Teflon | 350 Black lights |  | (39) | Continuous-flow chamber |
| 2011 | CESAM, CNRS | 4.2 | 4.3 | Stainless steel | xenon arc lamps |  | (40) | Pressure down to 10^-7^ atm  **Temperature range:** 15-60℃  <https://www.cesam.cnrs.fr/> |
| 2012 | Manchester Aerosol Chamber | 18 | 2.3 | FEP Teflon  Collapsible | Halogen/xenon arc | 0.09 | (41) | *J*_O1D_ = 1.23×10^-5^ s^-1^  Temperature: 288-313K  <http://www.cas.manchester.ac.uk/restools/aerosolchamber/> |
| 2013 | FORTH-ASC | 10 | 2.7 | Teflon |  | 0.01 | (42) | <http://laqs.iceht.forth.gr/> |
|  |  | 2×2 |  | Teflon | UV-equipped enclosure |  |  | Mobile chamber |
| 2014 | NCAR chamber | 10 |  | FEP Teflon | Dark conditions |  | (43) | Reagents input continuously |
| 2015 | ILMARI, UEF | 29 | 2 | FEP Teflon | Two types of lamps, spectra centered at 350 nm and 340 nm |  | (44) | <https://www3.uef.fi/en/web/ilmari> |
| 2016 | ISAC, CNRS | 2 | 5 | Teflon | UV and visible light sources |  | (45) | chamber sits a tank filled with liquid/material/chemicals |
|  | IASC | 27.2 | 2.1 |  | 125 UVA Lamps (Phillips Actinic BL TL 40W/10), 25 UVB Lamps (Phillips TL 40W/12 RS) | 0.36 |  | <http://www.ucc.ie/en/crac/facilities/iasc> |
| 2016 | Kyungpook Natl Univ. Korea | 7 |  | FEP Teflon |  | 0.17 | (46) | Temperature range: 291-306K |
| 2018 | ChAMBRe, INFN-Genova | 2.2 | 4.4 | Stainless steel |  |  | (47) | **Temperature range:** 18-25 °C  **Pressure range:** 10^-5^-1030 mbar  <https://labfisa.ge.infn.it/> |
| 1982 | Peking University | 1.2 | 5 | 50 μm FEP Teflon | Blacklight | 0.2 | (48) |  |
| 1989 | CRAES | 0.234 | 12.8 | 3.5 mm Quartz | Blacklight | 1.26 | (49) | Under vacuum |
| 1997 | CRAES | 3 | 3.9 | 50 μm FEP Teflon | Mercury lamp |  | (50) |  |
| 2001 | RCEES-CAS | 0.1 |  | FEP Teflon | Mercury lamp |  | (51) |  |
| 2002 | AIOFM-CAS | 0.233 | 22.4 | 4 mm Quartz | Blacklight |  | (52) |  |
| 2005 | Tsinghua University | 2 | 5 | 50 μm FEP Teflon | 40 Blacklight (40W) | 0.23 | (53) | Temperature (10-60 ℃, ±1.0 ℃); C_3_H_6_ wall loss rate: 0.0004 h^-1^ |
| 2007 | AIOFM-CAS | 0.850 | 5.8 | PE | Blacklight |  | (54) |  |
| 2009 | GIG-CAS | 30 | 2.1 | 54 μm FEP Teflon | Blacklight (60W) & Xenon arc lamp | 0.49 | (55) | Temperature (-10-40 ℃, ±1.0 ℃) |
| 2011 | IAP-CAS | 0.145-1.3 | 5.8-12 | 50 μm FEP Teflon | Blacklight | 0.44 | (56-58) |  |
| 2012 | RCEES-CAS | 3 | 3.7 | 75 μm FEP Teflon | Blacklight | 0.156 | (59, 60) | Temperature controlled; SO_2_ wall loss rate: 7.3×10^-8^ s^-1^ |
| 2013 | IC-CAS | 2×5 | 3.6 | 50 μm FEP Teflon | Left: 40 narrow-band black lamps, Right: 20 black lamps + 20 broad-band UV lamps | 0.153 & 0.139 | (61) | Temperature controlled (-10-40 ℃, ±0.5 ℃); C_3_H_6_ wall loss rates: 0.3×10^-4^ min^-1^ |
| 2013 | RCEES-CAS | 30 | 1.97 | 125 μm FEP Teflon | 120 UV light & Xenon arc lamp | 0.55 | (62-64) | Temperature (15-40 ℃, ±1.0 ℃); SO_2_ and VOCs wall loss rates: (4.52±0.11) ×10^-4^ and (2.20±0.39) ×10^-4^ min^-1^ |
| 2017 | Zhejiang University | 3 | 4.32 | 60 μm FEP Teflon | UV light | 0.12 | (65, 66) |  |
| 2019 | Shandong University | 3 |  | FEP Teflon |  |  | (67) |  |

*Reference publication year is used for some chambers

#### Table S2. The typical outdoor chambers around the world

| Year* | Place/Institute | Feature | Reference | Note/Website |
| --- | --- | --- | --- | --- |
| 1976 | UNC, Chapel Hill | 2×150 m^3^, FEP Teflon | (68-70) |  |
| 1985 |  | 25 m^3^, FEP Teflon | (71, 72) |  |
| 1995 |  | 190 m^3^, FEP Teflon | (73) |  |
| 2004 |  | 2×135 m^3^, FEP Teflon | (74, 75) | Replace the 150 m^3^ chamber |
| 2011 |  | 2×120 m^3^, FEP Teflon | (76) |  |
| 1979 | CSIRO, Sydney | 2×20m^3^, FEP Teflon | (77) |  |
| 1983 | OTC, Riverside | 50m^3^, Teflon | (7) |  |
| 1983 | Columbus Laboratories | 17.3 m^3^, FEP | (78, 79) |  |
| 1984 | ERT chamber | 60-80 m^3^, FEP 200A Teflon film, S/V: ~1.4 m^-1^ | (80, 81) |  |
| 1985 | Pasadena | 65 m^3^, 2-mil-thick FEP Teflon, S/V: 1.5 m^-l^ | (82) |  |
| 1992 | EPA | 9 m^3^, Teflon | (16) | Indoor+ mobile chamber, Mobile emission |
| 1995 | EUPHORE, Valencia | 2×200 m^3^, FEP, S/V: 1 m^-1^ | (83-86) | Ambient temperature (+ 0-6 K)  <http://www.ceam.es/WWWEUPHORE/home.htm> |
| 2000 | SAPHIR & PLUS, Jülich | 280-370m^3^, two layers 150 μm FEP | (87, 88) | Typical values for OH reactivity less than 1.5 s^-1^.  <https://www.fz-juelich.de/iek/iek-8/EN/Expertise/Infrastructure/SAPHIR/SAPHIR_node.html> |
| 2010 | UCLA | 24-34m^3^, 2-mil FEP | (89, 90) |  |
| 2012 | HELIOS, CNRS | 90 m^3^, FEP (250 μm) | (91) | Two types of irradiation (UV and Xenon lamps), *J*_NO2_=3.5-6.4×10^-3^ s^-1^, Wall loss of HCHO and VOCs rates < 2×10^-6^ s^-1^. SF_6_ dilution rate: 1.4×10^-5^ s^-1^  <https://helios-cnrs.org/> |
| 2014 | UF-APHOR, University of Florida | 2×52m^3^, FEP 125μm | (92) |  |
| 2021 | CRAES chamber | 56 m^3^, FEP 100 μm, S/V: ~1.55 m^-1^ | (93) |  |

*Reference publication year is used for some chambers

#### Table S3. Typical air pollutants monitored in smog chamber experimental systems and the corresponding measurement instruments or techniques

| Parameters/Pollutants | | Instruments/techniques | Reference or company | Importance |
| --- | --- | --- | --- | --- |
| Gas pollutants | VOCs | Gas chromatograph-mass spectrometer (GC-MS)  Proton transfer reaction mass spectrometry (PTR-MS)  Single photon ionization time-of-flight mass spectrometer (SPI-TofMS)  Chemical ionization spectroscopy (CIMS) | (94)  (95, 96)  (97-99) | Important precursors of secondary aerosol |
|  | Formaldehyde | Hantzsch instrument | (100) |  |
|  | SO_2_/SO_3_/H_2_SO_4_ | Pulsed fluorescence analyzer (e.g., Thermo Fisher Scientific 43i, Ecotech EC9850)  Tunable diode laser system  Chemical ionization spectroscopy (CIMS) | (101) |  |
|  | NH_3_ | Los Gatos research ammonia analyzer  Quantum cascade laser (QCL)  Quantum cascade tunable infrared laser direct absorption spectrometer (QC-TILDAS) | (102, 103) |  |
|  | NO_x_/NO_y_/NO_z_ | Chemiluminescence analyzer (e.g., Thermo Fisher Scientific 42i, Thermo Fisher Scientific 42i-NO_y_, Ecotech EC9841) |  | Required for studying nitrogen chemistry and characterizing oxidation capacity |
|  | O_3_ | UV photometric analyzer (e.g., Thermo Fisher Scientific 43i, Ecotech EC9810) |  | Required for characterizing oxidation capacity |
|  | HONO | Long-path absorption photometer (LOPAP)  Negative-ion proton-transfer chemical ionization mass spectrometric technique (NI-PT-CIMS) | (104)  (98, 105) |  |
|  | PAN | Gas chromatography-electron capture detector (GC-ECD)  Gas chromatography-pulsed discharge detector (GC-PDD) | (106)  (107) |  |
|  | Peroxide or hydroperoxide species | Chemical ionization spectroscopy (CIMS)  High-performance liquid chromatography (HPLC)  Peroxide-LOPAP | (97-99)  (108)  (109) | Oxidation products |
|  | CO | Gas filter correlation analyzer (e.g., Thermo Fisher Scientific 48i, Ecotech EC9830) |  | Important sink of OH radical |
| Aerosols | Number concentration | Condensation particle counter (CPC) | TSI Inc. | Important to quantify aerosol formation and growth |
|  | Size distribution | Scanning mobility particle spectrometer (SMPS)  Diethylene glycol (DEG) -SMPS  Neutral cluster and air ion spectrometer (NAIS)  Particle size magnifier (PSM)  Differential mobility analyzer train (DMA-train) | (110)  (111, 112) (113)  (114)  (115) |  |
|  | Ion size distribution | Neutral cluster and air ion spectrometer (NAIS) | (114) |  |
|  | Chemical composition | Ion chromatography (IC)  Gas chromatography-mass spectrometry (GC-MS)  Ultrahigh performance liquid chromatography (UHPLC)  Aerosol chemical speciation monitor (ACSM)/ Time-of-flight-ACSM (ToF-ACSM)  Aerosol mass spectrometry (AMS)/(ToF-AMS)  Thermal desorption aerosol GC-MS  Filter inlet for gas and aerosol (FIGAERO)-CIMS  Thermal desorption chemical ionization mass spectrometry (TDCIMS)CI-Orbitrap mass spectrometry  Liquid chromatography-Orbitrap mass spectrometry | (116, 117)  (118, 119)  (120-122)  (123, 124)  (125)  (126)  (127) | Important for reveal aerosol formation mechanisms |
|  | Optical properties | Cavity ring-down spectroscopy (CRDS)  Photoacoustic spectroscopy (PAS)  Integrating nephelometer (e.g., TSI 3563) | (128) | Important for understand environmental impacts |
|  | Hygroscopicity | Hygroscopicity tandem differential mobility analyzers (H-TDMA) | (129) |  |
|  | Morphology | Transmission electron microscopy (TEM) | (130) |  |
| Radicals | OH, HO_2_ | Fluorescence assay with gas expansion (FAGE)  Laser induced fluorescence (LIF) | (131) | Important for investigate oxidation mechanisms |
|  | NO_3_ | Cavity ring-down spectroscopy (CRDS)  Open-Path differential optical absorption spectroscopy (OP-DOAS)  Incoherent broad-band cavity enhanced absorption spectroscopy (IBB-CEAS) | (132)  (133) |  |
|  | sCI | Photoionization mass spectrometry | (134) |  |

#### Table S4. Background pollutant levels in some smog chambers

| Pollutant | Background level | Chamber volume and material | Reference | Note |
| --- | --- | --- | --- | --- |
| Ions | Positive: 133 ± 34 cm^−3^; Negative: 166 ± 30 cm^−3^ | 4.2 m^3^ stainless steel | (135) |  |
| Particles | <10 cm^−3^ | 24 m^3^ Teflon | (29) |  |
|  | < 1 cm^−3^ | 4.7 m^3^ Teflon | (39) |  |
|  | <0.2 cm^−3^ | 90 m^3^ Teflon | (136) | Below instrument detection limit |
|  | <0.1 cm^−3^ | 12 m^3^ Teflon | (27) |  |
|  | <10 cm^−3^ | 7 m^3^ Teflon | (46) | 10-1083.3 nm |
|  | ~ 2 cm^−3^ | 2.2 m^3^ stainless steel | (47) | Measured by an Optical Particle Counter (OPC) |
|  | < 1 cm^−3^ | 2×5 m^3^, Dual reactor chamber | (61) |  |
|  |  | 30 m^3^ Teflon | (137) | Below instrument detection limit |
|  | <10 cm^−3^ | 30 m^3^ Teflon | (64) |  |
| H_2_SO_4_ | <5×10^4^ cm^−3^ | 26 m^3^ stainless steel | (138) | Below instrument detection limit |
| SO_2_ | < 15 ppt | 26 m^3^ stainless steel | (138) | Below instrument detection limit |
|  | <1 ppb | 30 m^3^ Teflon | (64) |  |
| NOx | < 5ppb | 90 m^3^ Teflon | (22) |  |
|  | < 0.5ppb | 24 m^3^ Teflon | (29) |  |
|  | NO/NOx <100 ppt | 27 m^3^ Teflon | (25) |  |
|  | NO< 0.6ppb; NO_2_<0.6 ppb | 29 m^3^ Teflon | (44) |  |
|  | < 1ppb | 7 m^3^ Teflon | (46) |  |
|  | 1-2 ppb | 2.2 m^3^ stainless steel | (47) | Close to the analyzer sensitivity |
|  | <1 ppb | 2×5 m^3^, Dual reactor chamber | (61) |  |
|  |  | 30 m^3^ Teflon | (137) | Below instrument detection limit |
|  | <1 ppb | 30 m^3^ Teflon | (64) |  |
| HONO | 10 ppt | 24 m^3^ Teflon | (29) |  |
| O_3_ | < 1 ppb | 27 m^3^ Teflon | (25) | Below instrument detection limit |
|  | < 1 ppb | 7 m^3^ Teflon | (46) |  |
|  | 1-2 ppb | 2.2 m^3^ stainless steel | (47) | Close to the analyzer sensitivity |
|  | <1 ppb | 2×5 m^3^, Dual reactor chamber | (61) |  |
|  |  | 30 m^3^ Teflon | (137) | Below instrument detection limit |
|  | <1 ppb | 30 m^3^ Teflon | (64) |  |
| HCHO | < 5ppb | 90 m^3^ Teflon | (22) |  |
|  | <1ppb | 24 m^3^ Teflon | (29) |  |
| VOCs | NMHC< 17 ppb Cv | 24 m^3^ Teflon | (29) |  |
|  | (C5–C10) < 1 ppb | 7 m^3^ Teflon | (46) |  |
|  | (C1–C3) < 150 ppt | 26 m^3^ stainless steel | (138) |  |
|  | <1 ppb | 2×5 m^3^, Dual reactor chamber | (61) |  |
|  |  | 30 m^3^ Teflon | (137) | Below instrument detection limit |
| CO | < 6 ppb | 27 m^3^ Teflon | (25) | Below instrument detection limit |
|  | < 50 ppb | 90 m^3^ Teflon | (22) | Below instrument detection limit |
| CO_2_ | ~35 ppm | 12 m^3^ Teflon | (27) | Leak rate: 0.08% h^−1^ (i.e., 9 L h^−1^) |

#### Table S5. Wall loss rates in some smog chambers at room temperature

| Chamber volume and material | Gaseous pollutant wall loss rate (10^-4^ min^-1^) | | | Aerosol loss | | Reference |
| --- | --- | --- | --- | --- | --- | --- |
|  | NO | NO_2_ | O_3_ | Deposition constant (10^-3^ min^-1^) | Aerosol lifetime |  |
| 2 m^3^ Teflon |  |  |  | 4.72 |  | (23) |
| 2 m^3^ Teflon | 0.38 | 0.42 | 6.1 | 2.6 (100nm)  1.1(300nm) | <14 h | (53) |
| 2 m^3^ Teflon |  |  | 3.3 |  |  | (45) |
| 2.5 m^3^ Teflon | 7-20 | 4-20 | 12-24 |  |  | (24) |
| 3 m^3^ Teflon |  | 1.64 | 8.99 | 3.52 |  | (66) |
| 3 m^3^ Teflon |  |  | 1.38 |  |  | (59, 60) |
| 3 m^3^ Teflon |  |  |  | 2.84 |  | (67) |
| 5 m^3^ Teflon | 3.0-3.1 | 3.8-4.5 | 2.5-3.1 | 2.0-4.3 | 5.2-6.2 h | (61) |
| 7 m^3^ Teflon | 3.78 | 0.45 | 0.65 | 3.96 | 4 h (100 nm) | (46) |
| 9 m^3^ Teflon |  |  |  |  | 3-5 h | PSI chamber website |
| 10 m^3^ Teflon |  |  | 0.6 |  |  | (42)  <http://laqs.iceht.forth.gr/> |
| 18 m^3^ Teflon |  |  | 6.48 |  |  | (41)  <http://www.cas.manchester.ac.uk/restools/aerosolchamber/> |
| 24 m^3^ Teflon | 0.054 | 0.204 | 0.42 |  |  | (29) |
| 27 m^3^ Teflon |  |  | 2.4 |  | 12-15 h | (26) |
| 28 m^3^ Teflon |  |  |  |  | 74 h | (21) |
| 29 m^3^ Teflon |  |  | 0.39 |  | >8 h | (44)  <https://www3.uef.fi/en/web/ilmari> |
| 30 m^3^ Teflon | 1.41 | 1.39 | 1.31 | 1.33 (300 nm) |  | (55) |
| 30 m^3^ Teflon | 1.32 | 1.67 | 3.32 | 0.3-2.0 | 21.3 h | (64) |
| 60 m^3^ Teflon | 0-5.4 | 0-2 | 0.5-3  2-8 (sunlight/humid) |  |  | (81) |
| 90 m^3^ Teflon |  |  |  |  | 7 days | (22) |
| 90 m^3^ Teflon | < 1.2 | | |  |  | (91) |
| 200 m^3^ Teflon |  |  | 1.8 |  |  | (84) |
| 370 m^3^ Teflon |  |  | 1.8 | 1.2 | 14 h | (87) |
| 2 m^3^ stainless steel |  |  | 9-24 |  |  | (34) |
| 2.2 m^3^ stainless steel |  |  | 18±2.4 |  | 1-30 h | (47) |
| 4.2 m^3^ stainless steel |  |  | 20 |  | Up to 4 days | (40) |
| 6.1 m^3^ stainless steel coated with PFA |  |  | 6 |  |  | (10) |
| 84 m^3^ aluminum |  |  | 23 | 60 (Nucleation mode particles)  0.06 (10-1000nm) | 5 days | (14, 139)  <http://www.imk-aaf.kit.edu/73.php> |

# References

1. Morriss, FV, Bolze, C, Goodwin, JT*, et al.* Smog Experiments in Large Chambers. *Ind Eng Chem*. 1957; **49**(8): 1249-50.

2. Wu, CH, Niki, H. Methods for measuring NO2 photodissociation rate - application to smog chamber studies. *Environ Sci Technol*. 1975; **9**(1): 46-52.

3. Winer, AM, Darnall, KR, Atkinson, R*, et al.* Smog Chamber Study of the Correlation of Hydroxyl Radical Rate Constants with Ozone Formation. *Environ Sci Technol*. 1979; **13**(7): 822-6.

4. Carter, WPL, Winer, AM, Darnall, KR*, et al.* Smog chamber studies of temperature effects in photo-chemical smog. *Environ Sci Technol*. 1979; **13**(9): 1094-100.

5. Carter, WPL, Darnall, KR, Graham, RA*, et al.* Reactions of C2 and C4 alpha-hydroxy radicals with oxygen. *Journal of Physical Chemistry*. 1979; **83**(18): 2305-11.

6. Winer, AM, Graham, RA, Doyle, GJ*, et al.* An evacuable environmental chamber and solar simulator facility for the study of atmospheric photochemistry. *Adv Environ Sci Technol*. 1980; **10**: 461-511.

7. Dodge, MC. Chemical oxidant mechanisms for air quality modeling: critical review. *Atmos Environ*. 2000; **34**(12): 2103-30.

8. Carter, WPL, Atkinson, R. Computer Modeling Study of Incremental Hydrocarbon Reactivity. *Environ Sci Technol*. 1989; **23**(7): 864-80.

9. Meagher, JF, Olszyna, KJ, Simonaitis, R. Smog Chamber Study of H2O2 Formation in Ethene Nox and Propene NOx Mixtures. *Int J Chem Kinet*. 1990; **22**(7): 719-40.

10. Akimoto, H, Hoshino, M, Inoue, G*, et al.* Design and characterization of the evacuable and bakable photo-chemical smog chamber. *Environ Sci Technol*. 1979; **13**(4): 471-5.

11. Barnes, I, Becker, KH, Mihalopoulos, N. An FTIR product study of the photooxidation of dimethyl disulfide. *J Atmos Chem*. 1994; **18**(3): 267-89.

12. Evans, LF, Weeks, IA, Eccleston, AJ. A chamber study of photochemical smog in Melbourne, Australia - present and future. *Atmos Environ*. 1986; **20**(7): 1355-68.

13. Kamm, S, Mohler, O, Naumann, KH*, et al.* The heterogeneous reaction of ozone with soot aerosol. *Atmos Environ*. 1999; **33**(28): 4651-61.

14. Wagner, R, Bunz, H, Linke, C*, et al.* Chamber simulations of cloud chemistry: The AIDA chamber. *Nato Sci S Ss Iv Ear*. 2006; **62**: 67-+.

15. Wagner, R, Linke, C, Naumann, KH*, et al.* A review of optical measurements at the aerosol and cloud chamber AIDA. *J Quant Spectrosc Ra*. 2009; **110**(11): 930-49.

16. Kleindienst, TE, Smith, DF, Hudgens, EE*, et al.* The photooxidation of automobile emissions: measurement of the transformation products and their mutagenic activity. *Atmos Environ*. 1992; **26A**: 3039-53.

17. Edney, EO, Kleindienst, TE, Jaoui, M*, et al.* Formation of 2-methyl tetrols and 2-methylglyceric acid in secondary organic aerosol from laboratory irradiated isoprene/NO_x_/SO_2_/air mixtures and their detection in ambient PM_2.5_ samples collected in the eastern United States. *Atmos Environ*. 2005; **39**(29): 5281-9.

18. Mentel, TF, Bleilebens, D, Wahner, A. A study of nighttime nitrogen oxide oxidation in a large reaction chamber - The fate of NO2, N2O5, HNO3, and O3 at different humidities. *Atmos Environ*. 1996; **30**(23): 4007-20.

19. Bunce, NJ, Liu, L, Zhu, J*, et al.* Reaction of naphthalene and its derivatives with hydroxyl radicals in the gas phase. *Environ Sci Technol*. 1997; **31**(8): 2252-9.

20. Chu, B, Liu, Y, Li, J*, et al.* Decreasing effect and mechanism of FeSO_4_ seed particles on secondary organic aerosol in α-pinene photooxidation. *Environ Pollut*. 2014; **193**: 88-93.

21. Cocker, DR, Flagan, RC, Seinfeld, JH. State-of-the-Art Chamber Facility for Studying Atmospheric Aerosol Chemistry. *Environ Sci & Technol*. 2001; **35**(12): 2594-601.

22. Carter, WPL, Cocker, DR, Fitz, DR*, et al.* A new environmental chamber for evaluation of gas-phase chemical mechanisms and secondary aerosol formation. *Atmos Environ*. 2005; **39**(40): 7768-88.

23. Takekawa, H, Minoura, H, Yamazaki, S. Temperature dependence of secondary organic aerosol formation by photo-oxidation of hydrocarbons. *Atmos Environ*. 2003; **37**(24): 3413-24.

24. Bae, G-N, Choo, MK, 이승복*, et al.* Design and Performance Evaluation of the KIST Indoor Smog Chamber. *Journal of Korean Society for Atmospheric Environmnet*. 2003; **19**(4): 437-49.

25. Paulsen, D, Dommen, J, Kalberer, M*, et al.* Secondary organic aerosol formation by irradiation of 1,3,5-trimethylbenzene-NOx-H2O in a new reaction chamber for atmospheric chemistry and physics. *Environ Sci Technol*. 2005; **39**(8): 2668-78.

26. Metzger, A, Dommen, J, Gaeggeler, K*, et al.* Evaluation of 1,3,5 trimethylbenzene degradation in the detailed tropospheric chemistry mechanism, MCMv3.1, using environmental chamber data. *Atmos Chem Phys*. 2008; **8**(21): 6453-68.

27. Platt, SM, El Haddad, I, Zardini, AA*, et al.* Secondary organic aerosol formation from gasoline vehicle emissions in a new mobile environmental reaction chamber. *Atmos Chem Phys*. 2013; **13**(18): 9141-58.

28. Hynes, RG, Angove, DE, Saunders, SM*, et al.* Evaluation of two MCM v3.1 alkene mechanisms using indoor environmental chamber data. *Atmos Environ*. 2005; **39**(38): 7251-62.

29. White, S, Angove, D, Li, K*, et al.* Development of a new smog chamber for studying the impact of different UV lamps on SAPRC chemical mechanism predictions and aerosol formation. *Environ Chem*. 2018; **15**(3): 171-82.

30. Kirkby, J, Curtius, J, Almeida, J*, et al.* Role of sulphuric acid, ammonia and galactic cosmic rays in atmospheric aerosol nucleation. *Nature*. 2011; **476**(7361): 429-33.

31. Duplissy, J, Merikanto, J, Franchin, A*, et al.* Effect of ions on sulfuric acid-water binary particle formation: 2. Experimental data and comparison with QC-normalized classical nucleation theory. *J Geophys Res-Atmos*. 2016; **121**(4): 1752-75.

32. Stanier, CO, Pathak, RK, Pandis, SN. Measurements of the volatility of aerosols from alpha-piniene ozonolysis. *Environ Sci Technol*. 2007; **41**(8): 2756-63.

33. Robinson, AL, Donahue, NM, Shrivastava, MK*, et al.* Rethinking organic aerosols: Semivolatile emissions and photochemical aging. *Science*. 2007; **315**(5816): 1259-62.

34. Glowacki, DR, Goddard, A, Hemavibool, K*, et al.* Design of and initial results from a Highly Instrumented Reactor for Atmospheric Chemistry (HIRAC). *Atmos Chem Phys*. 2007; **7**(20): 5371-90.

35. Temime, B, Healy, RM, Wenger, JC. A denuder-filter sampling technique for the detection of gas and particle phase carbonyl compounds. *Environ Sci Technol*. 2007; **41**(18): 6514-20.

36. Healy, RM, Wenger, JC, Metzger, A*, et al.* Gas/particle partitioning of carbonyls in the photooxidation of isoprene and 1,3,5-trimethylbenzene. *Atmos Chem Phys*. 2008; **8**(12): 3215-30.

37. Mutzel, A, Poulain, L, Berndt, T*, et al.* Highly Oxidized Multifunctional Organic Compounds Observed in Tropospheric Particles: A Field and Laboratory Study. *Environ Sci Technol*. 2015; **49**(13): 7754-61.

38. Iinuma, Y, Muller, C, Berndt, T*, et al.* Evidence for the existence of organosulfates from beta-pinene ozonolysis in ambient secondary organic aerosol. *Environ Sci Technol*. 2007; **41**(19): 6678-83.

39. King, SM, Rosenoern, T, Shilling, JE*, et al.* Increased cloud activation potential of secondary organic aerosol for atmospheric mass loadings. *Atmos Chem Phys*. 2009; **9**(9): 2959-71.

40. Wang, J, Doussin, J, Perrier, S*, et al.* Design of a new multi-phase experimental simulation chamber for atmospheric photosmog, aerosol and cloud chemistry research. *Atmo Meas Tech*. 2011; **4**(11): 2465.

41. Alfarra, MR, Hamilton, JF, Wyche, KP*, et al.* The effect of photochemical ageing and initial precursor concentration on the composition and hygroscopic properties of beta-caryophyllene secondary organic aerosol. *Atmos Chem Phys*. 2012; **12**(14): 6417-36.

42. Kostenidou, E, Kaltsonoudis, C, Tsiflikiotou, M*, et al.* Burning of olive tree branches: a major organic aerosol source in the Mediterranean. *Atmos Chem Phys*. 2013; **13**(17): 8797-811.

43. Fry, JL, Draper, DC, Barsanti, KC*, et al.* Secondary Organic Aerosol Formation and Organic Nitrate Yield from NO3 Oxidation of Biogenic Hydrocarbons. *Environ Sci Technol*. 2014; **48**(20): 11944-53.

44. Leskinen, A, Yli-Pirila, P, Kuuspalo, K*, et al.* Characterization and testing of a new environmental chamber. *Atmo Meas Tech*. 2015; **8**(6): 2267-78.

45. Bernard, F, Ciuraru, R, Boreave, A*, et al.* Photosensitized Formation of Secondary Organic Aerosols above the Air/Water Interface. *Environ Sci Technol*. 2016; **50**(16): 8678-86.

46. Bin Babar, Z, Park, J-H, Kang, J*, et al.* Characterization of a Smog Chamber for Studying Formation and Physicochemical Properties of Secondary Organic Aerosol. *Aerosol Air Qual Res*. 2016; **16**(12): 3102-13.

47. Massabo, D, Danelli, SG, Brotto, P*, et al.* ChAMBRe: a new atmospheric simulation chamber for aerosol modelling and bio-aerosol research. *Atmo Meas Tech*. 2018; **11**(10): 5885-900.

48. Tang, X, Bi, M, Li, J*, et al.* Trial production and performance experiment of photochemical smog chamber. *Environ Chem*. 1982(05): 344-51.

49. Wang, W, Wang, D, Liu, Y*, et al.* The Structure and Characteristics of the evacuable photochemical smog chamber. *China Environ Sci*. 1989; **9**(4): 304-10.

50. Wang, W, Shu, Y, Li, J. Photochemical degradation of PAHS on smoke particles in atmosphere. *China Environ Sci*. 1997(02): 2-7.

51. Wu, H, Mu, Y, Zhang, X*, et al.* Rate constants for reactions of hydroxyl radicals with a series of alcohols by relative rate method. *Acta Scientiae Circumstantiae*. 2001(05): 525-9.

52. Nie, J, Qin, M, Yang, Y*, et al.* The structure and performance of a kind of photo chemical smog chamber. *Journal of Atomic and Molecular Physics*. 2002(02): 186-90.

53. Wu, S, Lu, ZF, Hao, JM*, et al.* Construction and characterization of an atmospheric simulation smog chamber. *Adv Atmos Sci*. 2007; **24**(2): 250-8.

54. Hao, L-q, Wang, Z-y, Huang, M-q*, et al.* Effects of seed aerosols on the growth of secondary organic aerosols from the photooxidation of toluene. *J Environ Sci*. 2007; **19**(6): 704-8.

55. Wang, X, Liu, T, Bernard, F*, et al.* Design and characterization of a smog chamber for studying gas-phase chemical mechanisms and aerosol formation. *Atmo Meas Tech*. 2014; **7**(1): 301-13.

56. Ge, S, Xu, Y, Jia, L. Secondary organic aerosol formation from propylene irradiations in a chamber study. *Atmos Environ*. 2017; **157**: 146-55.

57. Jia, L, Xu, Y. Different roles of water in secondary organic aerosol formation from toluene and isoprene. *Atmos Chem Phys*. 2018; **18**(11): 8137-54.

58. Jia, L, Xu, Y, Shi, Y. Characterization of Photochemical Smog Chamber and Initial Experiments. *Environmental Science*. 2011; **32**(02): 351-61.

59. Song, M, Zhang, C, Wu, H*, et al.* The influence of UV-light irradiation and stable Criegee intermediate scavengers on secondary organic aerosol formation from isoprene ozonolysis. *Atmos Environ*. 2018; **191**: 116-25.

60. Song, M, Zhang, C, Wu, H*, et al.* The influence of OH concentration on SOA formation from isoprene photooxidation. *Sci Total Environ*. 2019; **650**: 951-7.

61. Wang, W-G, Li, K, Zhou, L*, et al.* Evaluation and Application of Dual-Reactor Chamber for Studying Atmospheric Oxidation Processes and Mechanisms. *Acta Physico-Chimica Sinica*. 2015; **31**(7): 1251-9.

62. Chen, T, Liu, Y, Chu, B*, et al.* Differences of the oxidation process and secondary organic aerosol formation at low and high precursor concentrations. *J Environ Sci*. 2019; **79**: 256-63.

63. Chen, T, Liu, Y, Liu, C*, et al.* Important role of aromatic hydrocarbons in SOA formation from unburned gasoline vapor. *Atmos Environ*. 2019; **201**: 101-9.

64. Chen, T, Liu, Y, Ma, Q*, et al.* Significant source of secondary aerosol: Formation from gasoline evaporative emissions in the presence of SO_2_ and NH_3_. *Atmos Chem Phys*. 2019; **19**(12): 8063-81.

65. Chen, L, Zheng, C, Gao, X*, et al.* Ozone and Secondary Organic Aerosol Formation of Toluene/NOx Irradiations under Complex Pollution Scenarios. *Aerosol Air Qual Res*. 2017; **17**(7): 1760-71.

66. Li, K, Chen, L, Han, K*, et al.* Smog chamber study on aging of combustion soot in isoprene/SO2/NOx system: Changes of mass, size, effective density, morphology and mixing state. *Atmospheric Research*. 2017; **184**: 139-48.

67. Liu, S, Tsona, NT, Zhang, Q*, et al.* Influence of relative humidity on cyclohexene SOA formation from OH photooxidation. *Chemosphere*. 2019; **231**: 478-86.

68. Jeffries, H, Fox, D, Kamens, R. Outdoor smog chamber studies - light effects relative to indoor chambers. *Environ Sci & Technol*. 1976; **10**(10): 1006-11.

69. Yu, JZ, Jeffries, HE, Lelacheur, RM. Identifying Airborne Carbonyl-Compounds in Isoprene Atmospheric Photooxidation Products by Their Pfbha Oximes Using Gas-Chromatography Ion-Trap Mass-Spectrometry. *Environ Sci Technol*. 1995; **29**(8): 1923-32.

70. Liu, XY, Jeffries, HE, Sexton, KG. Hydroxyl radical and ozone initiated photochemical reactions of 1,3-butadiene. *Atmos Environ*. 1999; **33**(18): 3005-22.

71. Kamens, RM, Rives, GD, Perry, JM*, et al.* Mutagenic Changes in Dilute Wood Smoke as It Ages and Reacts with Ozone and Nitrogen-Dioxide - an Outdoor Chamber Study. *Environ Sci Technol*. 1984; **18**(7): 523-30.

72. Saucy, DA, Kamens, RM, Linton, RW. An Aerosol Injection and Outdoor Chamber System for the Study of Atmospheric Gas-Particle Reactions. *Atmos Environ*. 1983; **17**(12): 2617-24.

73. Kamens, R, Odum, J, Fan, ZH. Some Observations on Times to Equilibrium for Semivolatile Polycyclic Aromatic-Hydrocarbons. *Environ Sci Technol*. 1995; **29**(1): 43-50.

74. Lee, S, Jang, M, Kamens, RM. SOA formation from the photooxidation of α-pinene in the presence of freshly emitted diesel soot exhaust. *Atmos Environ*. 2004; **38**(16): 2597-605.

75. Zhang, H, Surratt, JD, Lin, YH*, et al.* Effect of relative humidity on SOA formation from isoprene/NO photooxidation: enhancement of 2-methylglyceric acid and its corresponding oligoesters under dry conditions. *Atmos Chem Phys*. 2011; **11**(13): 6411-24.

76. Ebersviller, S, Lichtveld, K, Sexton, KG*, et al.* Gaseous VOCs rapidly modify particulate matter and its biological effects - Part 2: Complex urban VOCs and model PM. *Atmos Chem Phys*. 2012; **12**(24): 12293-312.

77. Hess, GD, Carnovale, F, Cope, ME*, et al.* The evaluation of some photochemical smog reaction mechanisms—I. Temperature and initial composition effects. *Atmospheric Environment Part A General Topics*. 1992; **26**(4): 625-41.

78. Spicer, CW, Sverdrup, GM, Kuhlman, MR. Smog chamber studies of NOx chemistry in power-plant plumes. *Atmos Environ*. 1981; **15**(10-1): 2353-65.

79. Spicer, CW. Smog chamber studies of NOx transformation rate and nitrate precursor relationships. *Environ Sci Technol*. 1983; **17**(2): 112-20.

80. Grosjean, D. Photooxidation of Methyl Sulfide, Ethyl Sulfide, and Methanethiol. *Environ Sci Technol*. 1984; **18**(6): 460-8.

81. Grosjean, D. Wall loss of gaseous-pollutants in outdoor teflon chambers. *Environ Sci & Technol*. 1985; **19**(11): 1059-65.

82. Leone, JA, Flagan, RC, Grosjean, D*, et al.* An outdoor smog chamber and modeling study of toluene-NOx photooxidation. *Int J Chem Kinet*. 1985; **17**(2): 177-216.

83. Bloss, C, Wagner, V, Bonzanini, A*, et al.* Evaluation of detailed aromatic mechanisms (MCMv3 and MCMv3.1) against environmental chamber data. *Atmos Chem Phys*. 2005; **5**: 623-39.

84. Bloss, C, Wagner, V, Jenkin, ME*, et al.* Development of a detailed chemical mechanism (MCMv3.1) for the atmospheric oxidation of aromatic hydrocarbons. *Atmos Chem Phys*. 2005; **5**(3): 641-64.

85. Zador, J, Wagner, V, Wirtz, K*, et al.* Quantitative assessment of uncertainties for a model of tropospheric ethene oxidation using the European Photoreactor (EUPHORE). *Atmos Environ*. 2005; **39**(15): 2805-17.

86. Zador, J, Turanyi, T, Wirtz, K*, et al.* Measurement and investigation of chamber radical sources in the European Photoreactor (EUPHORE). *J Atmos Chem*. 2006; **55**(2): 147-66.

87. Rohrer, F, Bohn, B, Brauers, T*, et al.* Characterisation of the photolytic HONO-source in the atmosphere simulation chamber SAPHIR. *Atmos Chem Phys*. 2005; **5**: 2189-201.

88. Hohaus, T, Kuhn, U, Andres, S*, et al.* A new plant chamber facility, PLUS, coupled to the atmosphere simulation chamber SAPHIR. *Atmo Meas Tech*. 2016; **9**(3): 1247-59.

89. Chung, A, Lall, AA, Paulson, SE. Particulate emissions by a small non-road diesel engine: Biodiesel and diesel characterization and mass measurements using the extended idealized aggregates theory. *Atmos Environ*. 2008; **42**(9): 2129-40.

90. Kim, H, Barkey, B, Paulson, SE. Real refractive indices of alpha- and beta-pinene and toluene secondary organic aerosols generated from ozonolysis and photo-oxidation. *J Geophys Res-Atmos*. 2010; **115**.

91. Ren, YG, Grosselin, B, Daele, V*, et al.* Investigation of the reaction of ozone with isoprene, methacrolein and methyl vinyl ketone using the HELIOS chamber. *Faraday Discuss*. 2017; **200**: 289-311.

92. Im, Y, Jang, M, Beardsley, RL. Simulation of aromatic SOA formation using the lumping model integrated with explicit gas-phase kinetic mechanisms and aerosol-phase reactions. *Atmos Chem Phys*. 2014; **14**(8): 4013-27.

93. Li, J, Li, H, Wang, X*, et al.* A large-scale outdoor atmospheric simulation smog chamber for studying atmospheric photochemical processes: Characterization and preliminary application. *J Environ Sci*. 2021; **102**: 185-97.

94. de Gouw, J, Warneke, C. Measurements of volatile organic compounds in the earths atmosphere using proton-transfer-reaction mass spectrometry. *Mass Spectrom Rev*. 2007; **26**(2): 223-57.

95. Gao, W, Tan, GB, Hong, Y*, et al.* Development of portable single photon ionization time-of-flight mass spectrometer combined with membrane inlet. *Int J Mass Spectrom*. 2013; **334**: 8-12.

96. Yamamoto, Y, Kambe, Y, Yamada, H*, et al.* Measurement of volatile organic compounds in vehicle exhaust using single-photon ionization time-of-flight mass spectrometry. *Analytical Sciences the International Journal of the Japan Society for Analytical Chemistry*. 2012; **28**(4): 385-90.

97. Crounse, JD, McKinney, KA, Kwan, AJ*, et al.* Measurement of Gas-Phase Hydroperoxides by Chemical Ionization Mass Spectrometry. *Analytical Chemistry*. 2006; **78**(19): 6726-32.

98. Veres, P, Roberts, JM, Warneke, C*, et al.* Development of negative-ion proton-transfer chemical-ionization mass spectrometry (NI-PT-CIMS) for the measurement of gas-phase organic acids in the atmosphere. *Int J Mass Spectrom*. 2008; **274**(1-3): 48-55.

99. Jokinen, T, Sipila, M, Junninen, H*, et al.* Atmospheric sulphuric acid and neutral cluster measurements using CI-APi-TOF. *Atmos Chem Phys*. 2012; **12**(9): 4117-25.

100. Kelly, TJ, Fortune, CR. Continuous Monitoring of Gaseous Formaldehyde Using an Improved Fluorescence Approach. *Int J Environ Anal Chem*. 1994; **54**(4): 249-63.

101. Berkoff, TA, Wormhoudt, J, Miake-Lye, RC. Measurement of SO2 and SO3 using a tunable diode laser system. In: VoDinh, T, Spellicy, RL (eds.). *Environmental Monitoring and Remediation Technologies*1999. 686-93.

102. Kosterev, AA, Curl, RF, Tittel, FK*, et al.* Transportable automated ammonia sensor based on a pulsed thermoelectrically cooled quantum-cascade distributed feedback laser. *Applied Optics*. 2002; **41**(3): 573-8.

103. Ellis, RA, Murphy, JG, Pattey, E*, et al.* Characterizing a Quantum Cascade Tunable Infrared Laser Differential Absorption Spectrometer (QC-TILDAS) for measurements of atmospheric ammonia. *Atmos Meas Tech*. 2010; **3**(2): 397-406.

104. Kleffmann, J, Heland, J, Kurtenbach, R*, et al.* A new instrument (LOPAP) for the detection of nitrous acid (HONO). *Environ Sci Pollut R*. 2002: 48-54.

105. Roberts, JM, Veres, P, Warneke, C*, et al.* Measurement of HONO, HNCO, and other inorganic acids by negative-ion proton-transfer chemical-ionization mass spectrometry (NI-PT-CIMS): application to biomass burning emissions. *Atmo Meas Tech*. 2010; **3**(4): 981-90.

106. Zhang, G, Mu, Y, Zhou, L*, et al.* Summertime distributions of peroxyacetyl nitrate (PAN) and peroxypropionyl nitrate (PPN) in Beijing: Understanding the sources and major sink of PAN. *Atmos Environ*. 2015; **103**: 289-96.

107. Zhang, L, Jaffe, DA, Gao, X*, et al.* A quantification method for peroxyacetyl nitrate (PAN) using gas chromatography (GC) with a non-radioactive pulsed discharge detector (PDD). *Atmos Environ*. 2018; **179**: 23-30.

108. Lee, M, Noone, BC, Osullivan, D*, et al.* Method for the Collection and Hplc Analysis of Hydrogen-Peroxide and C-1 and C-2 Hydroperoxides in the Atmosphere. *Journal of Atmospheric and Oceanic Technology*. 1995; **12**(5): 1060-70.

109. Mertes, P, Pfaffenberger, L, Dommen, J*, et al.* Development of a sensitive long path absorption photometer to quantify peroxides in aerosol particles (Peroxide-LOPAP). *Atmos Meas Tech*. 2012; **5**(10): 2339-48.

110. Wang, SC, Flagan, RC. Scanning Electrical Mobility Spectrometer. *Aerosol Sci Tech*. 1990; **13**(2): 230-40.

111. Jiang, J, Chen, M, Kuang, C*, et al.* Electrical Mobility Spectrometer Using a Diethylene Glycol Condensation Particle Counter for Measurement of Aerosol Size Distributions Down to 1 nm. *Aerosol Sci Technol*. 2011; **45**(4): 510-21.

112. Cai, RL, Chen, DR, Hao, JM*, et al.* A miniature cylindrical differential mobility analyzer for sub-3 nm particle sizing. *J Aerosol Sci*. 2017; **106**: 111-9.

113. Liu, JQ, Jiang, JK, Zhang, Q*, et al.* A spectrometer for measuring particle size distributions in the range of 3 nm to 10 mu m. *Front Env Sci Eng*. 2016; **10**(1): 63-72.

114. Mirme, S, Mirme, A. The mathematical principles and design of the NAIS - a spectrometer for the measurement of cluster ion and nanometer aerosol size distributions. *Atmo Meas Tech*. 2013; **6**(4): 1061-71.

115. Vanhanen, J, Mikkilä, J, Lehtipalo, K*, et al.* Particle size magnifier for Nano-CN detection. *Aerosol Sci Tech*. 2011; **45**(4): 533-42.

116. Ng, NL, Herndon, SC, Trimborn, A*, et al.* An Aerosol Chemical Speciation Monitor (ACSM) for routine monitoring of the composition and mass concentrations of ambient aerosol. *Aerosol Sci Tech*. 2011; **45**(7): 770-84.

117. Frohlich, R, Cubison, MJ, Slowik, JG*, et al.* The ToF-ACSM: a portable aerosol chemical speciation monitor with TOFMS detection. *Atmo Meas Tech*. 2013; **6**(11): 3225-41.

118. Baltensperger, U, Chirico, R, DeCarlo, PF*, et al.* Recent Developments in the Mass Spectrometry of Atmospheric Aerosols. *Eur J Mass Spectrom*. 2010; **16**(3): 389-95.

119. Drewnick, F, Hings, SS, DeCarlo, P*, et al.* A new time-of-flight aerosol mass spectrometer (TOF-AMS) - Instrument description and first field deployment. *Aerosol Sci Tech*. 2005; **39**(7): 637-58.

120. Williams, BJ, Goldstein, AH, Kreisberg, NM*, et al.* An in-situ instrument for speciated organic composition of atmospheric aerosols: Thermal Desorption Aerosol GC/MS-FID (TAG). *Aerosol Sci Tech*. 2006; **40**(8): 627-38.

121. Ren, HX, Xue, M, An, ZJ*, et al.* Quartz filter-based thermal desorption gas chromatography mass spectrometry for in-situ molecular level measurement of ambient organic aerosols. *J Chromatogr*. 2019; **1589**: 141-8.

122. An, ZJ, Ren, HX, Xue, M*, et al.* Comprehensive two-dimensional gas chromatography mass spectrometry with a solid-state thermal modulator for in-situ speciated measurement of organic aerosols. *J Chromatogr*. 2020; **1625**.

123. Lopez-Hilfiker, FD, Mohr, C, Ehn, M*, et al.* A novel method for online analysis of gas and particle composition: description and evaluation of a Filter Inlet for Gases and AEROsols (FIGAERO). *Atmo Meas Tech*. 2014; **7**(4): 983-1001.

124. Lee, BH, Lopez-Hilfiker, FD, Mohr, C*, et al.* An Iodide-Adduct High-Resolution Time-of-Flight Chemical-Ionization Mass Spectrometer: Application to Atmospheric Inorganic and Organic Compounds. *Environ Sci Technol*. 2014; **48**(11): 6309-17.

125. Smith, JN, Moore, KF, McMurry, PH*, et al.* Atmospheric measurements of sub-20 nm diameter particle chemical composition by thermal desorption chemical ionization mass spectrometry. *Aerosol Sci Tech*. 2004; **38**(2): 100-10.

126. Nizkorodov, SA, Laskin, J, Laskin, A. Molecular chemistry of organic aerosols through the application of high resolution mass spectrometry. *Phys Chem Chem Phys*. 2011; **13**(9): 3612-29.

127. Wang, K, Huang, RJ, Brüggemann, M*, et al.* Urban organic aerosol composition in Eastern China differs from North to South: Molecular insight from a liquid chromatography-Orbitrap mass spectrometry study. *Atmos Chem Phys Discuss*. 2019; **2019**: 1-30.

128. Moise, T, Flores, JM, Rudich, Y. Optical Properties of Secondary Organic Aerosols and Their Changes by Chemical Processes. *Chem Rev*. 2015; **115**(10): 4400-39.

129. Duplissy, J, Gysel, M, Sjogren, S*, et al.* Intercomparison study of six HTDMAs: results and recommendations. *Atmos Meas Tech*. 2009; **2**(2): 363-78.

130. DeCarlo, PF, Slowik, JG, Worsnop, DR*, et al.* Particle Morphology and Density Characterization by Combined Mobility and Aerodynamic Diameter Measurements. Part 1: Theory. *Aerosol Sci Tech*. 2004; **38**(12): 1185-205.

131. Hard, TM, George, LA, Obrien, RJ. Fage Determination of Tropospheric Ho and Ho2. *J Atmos Sci*. 1995; **52**(19): 3354-72.

132. Ventrillard-Courtillot, I, Sciamma O’Brien, E, Kassi, S*, et al.* Incoherent broad-band cavity-enhanced absorption spectroscopy for simultaneous trace measurements of NO2 and NO3 with a LED source. *Applied Physics B*. 2010; **101**(3): 661-9.

133. Dorn, HP, Apodaca, RL, Ball, SM*, et al.* Intercomparison of NO_3_ radical detection instruments in the atmosphere simulation chamber SAPHIR. *Atmos Meas Tech*. 2013; **6**(5): 1111-40.

134. Welz, O, Savee, JD, Osborn, DL*, et al.* Direct Kinetic Measurements of Criegee Intermediate (CH2OO) Formed by Reaction of CH2I with O2. *Science*. 2012; **335**(6065): 204-7.

135. Boulon, J, Sellegri, K, Katrib, Y*, et al.* Sub-3 nm Particles Detection in a Large Photoreactor Background: Possible Implications for New Particles Formation Studies in a Smog Chamber. *Aerosol Sci Tech*. 2013; **47**(2): 153-7.

136. Nakao, S, Clark, C, Tang, P*, et al.* Secondary organic aerosol formation from phenolic compounds in the absence of NOx. *Atmos Chem Phys*. 2011; **11**(20): 10649-60.

137. Zhang, Y, Deng, W, Hu, Q*, et al.* Comparison between idling and cruising gasoline vehicles in primary emissions and secondary organic aerosol formation during photochemical ageing. *Sci Total Environ*. 2020; **722**: 137934.

138. Kirkby, J, Duplissy, J, Sengupta, K*, et al.* Ion-induced nucleation of pure biogenic particles. *Nature*. 2016; **533**(7604): 521-6.

139. Saathoff, H, Naumann, KH, Mohler, O*, et al.* Temperature dependence of yields of secondary organic aerosols from the ozonolysis of alpha-pinene and limonene. *Atmos Chem Phys*. 2009; **9**(5): 1551-77.
